# DISTRIBUTIONAL IMPLICATIONS OF A NATIONAL CO_2_ TAX IN THE U.S. ACROSS INCOME CLASSES AND REGIONS: A MULTI-MODEL OVERVIEW

**DOI:** 10.1142/S2010007818400043

**Published:** 2018-03-20

**Authors:** JUSTIN CARON, JEFFERSON COLE, RICHARD GOETTLE, CHIKARA ONDA, JAMES MCFARLAND, JARED WOOLLACOTT

**Affiliations:** Massachusetts Institute of Technology, 77 Massachusetts Avenue Cambridge, MA 02139, USA; HEC Montréal, 3000 chemin de la Côte-Sainte-Catherine Montréal, QC, H3T 2A7, Canada; U.S. Environmental Protection Agency, 1200 Pennsylvania Avenue NW, Washington, DC 20460, USA; Northeastern University, 360 Huntington Ave Boston, MA 02115, USA; Dale Jorgenson Associates, 433 NH Route 119 East Fitzwilliam, NH 03447, USA; Stanford University, Emmett Interdisciplinary Program in Environment and Resources, 473 Via Ortega, Y2E2 Suite 226, Stanford, CA 94305, USA; U.S. Environmental Protection Agency, 1200 Pennsylvania Avenue NW, Washington, DC 20460, USA; RTI International, 3040 E. Cornwallis Rd. Durham, NC 27709, USA

**Keywords:** Climate policy, CO_2_ tax, carbon tax, distributional impacts, equity, progressivity, household welfare, double-dividends, model comparison, computable general equilibrium modeling

## Abstract

This paper presents a multi-model assessment of the distributional impacts of carbon pricing. A set of harmonized representative CO_2_ taxes and tax revenue recycling schemes is implemented in five large-scale economy-wide general equilibrium models. Recycling schemes include various combinations of uniform transfers to households and labor and capital income tax reductions. Particular focus is put on equity — the distribution of impacts across household incomes — and efficiency, evaluated in terms of household welfare. Despite important differences in the assumptions underlying the models, we find general agreement regarding the ranking of recycling schemes in terms of both efficiency and equity. All models identify a clear trade-off between efficient but regressive capital tax reductions and progressive but costly uniform transfers to households; all agree upon the inferiority of labor tax reductions in terms of welfare efficiency; and all agree that different combinations of capital tax reductions and household transfers can be used to balance efficiency and distributional concerns. A subset of the models go further and find that equity concerns, particularly regarding the impact of the tax on low income households, can be alleviated without sacrificing much of the double-dividend benefits offered by capital tax rebates. There is, however, less agreement regarding the progressivity of CO_2_ taxation net of revenue recycling. Regionally, the models agree that abatement and welfare impacts will vary considerably across regions of the U.S. and generally agree on their broad geographical distribution. There is, however, little agreement regarding the regions which would profit more from the various recycling schemes.

## 1. Introduction

Climate change mitigation policies that aim at putting a price on carbon emissions can have differing effects on household welfare, depending on income level and region. Households will be affected through changes in the prices of carbon-intensive goods, as well as changes in consumption behavior, production technology, and incomes. Welfare impacts of carbon taxation are also highly dependent on the way in which revenues from the tax are recycled, which also has varying distributional implications. These need to be taken into consideration by policy-makers in addition to efficiency considerations.

This paper presents the results from a model comparison exercise involving five computable general equilibrium (CGE) economic models capable of simulating distributional impacts from among a larger set of models participating in the Energy Modeling Forum (EMF) 32 model comparison exercise (Fawcett *et al.*, 2018). Particular attention is given to identifying the trade-off between maximizing aggregate economic efficiency and minimizing inequality in impacts. The models differ in many dimensions including the representation of the electricity system, the dynamic behavior of economic agents and underlying datasets used for calibration, but each captures the general equilibrium impacts of carbon pricing on household welfare. The models have been used to simulate a standardized set of CO_2_ tax scenarios with various revenue recycling schemes including lump-sum transfers to households as well as rebates to capital and labor taxes. We investigate distributional impacts using various measures of inequality and progressivity, taking care to standardize results across models.

This study is motivated by the following questions:

- What are the distributional impacts of carbon pricing?
- How do various revenue recycling schemes affect these impacts?
- How important is the trade-off between efficiency and equity?
- Can the lowest income households be compensated? At what cost?
- What is the distribution of impacts across regions of the U.S.?

We build upon a literature on the distributional impacts of climate policy and environmental regulation more broadly dating back to the 1970s (Parry *et al.*, 2006). Early studies typically rely on consumption data and input–output tables to determine the emissions embodied in the consumption of income groups. Examples include estimations of the compliance costs of a variety of policies including the Clean Air Act (Gianessi *et al.*, 1979; Robinson, 1985), a gasoline tax (Poterba, 1991), and a carbon tax (Bull *et al.*, 1994). These input–output analyses, and subsequent analyses on more recent policy proposals find that carbon pricing affects low-income households proportionally more than high-income households (i.e., is regressive), given the relatively more emissions-intense consumption bundles of lower income groups (e.g., Grainger and Kolstad, 2010).

However, such analyses are unable to consider changes to consumption behavior and the structure of the economy in response to tax-driven changes in goods and input prices, nor do they account for changes in factor income such as wages and returns to capital and fossil fuel resources. The literature based on CGE models, which attempt to capture all interactions and feedbacks across all markets, qualifies the regressive distributional impacts of carbon pricing. Using the USREP model, Rausch *et al.* (2010) find that, ignoring the issue of allowance allocation and revenue recycling, carbon taxation is proportional to mildly progressive. This result differs from previous work due to a strong reduction in capital income which affects higher-income households and the inflation-indexing of government transfers, which shield lower-income households from increased costs. They find similar results using a highly disaggregated household decision-making model based on the Consumer Expenditure Survey (Rausch *et al.*, 2011). The inflation-indexing of government transfers also contributes to the progressivity of carbon pricing in more recent literature (Cronin *et al.*, 2017).

While the literature has not seemingly reached a consensus regarding the progressivity or regressivity of carbon taxation itself, there is broad agreement that distributional impacts are ultimately largely driven by what is done with the collected revenue. The importance of revenue use on incidence was discussed as early as Metcalf (1999). The literature has identified that large efficiency gains could be provided by “tax swaps”, where the carbon tax revenue is used to reduce other distortionary taxes (Goulder *et al.*, 1999; Goulder, 2013). Since then, several studies have identified a trade-off between efficiency and equity, with corporate or capital income tax reductions being regressive but efficient (sometimes leading to a net increase in welfare, or a “strong double-dividend”), and lump-sum revenue recycling being progressive but inefficient (Dinan and Rogers, 2002; Burtraw *et al.*, 2009; Parry and Williams, 2010).

The present study updates, confirms and quantifies these findings, providing the first cross-model assessment of robust results coming out of five state-of-the-art economy-wide models which capture many of the channels through which carbon taxation affects households. We also provide results for a large set of CO_2_ price paths and revenue recycling schemes.

We clearly confirm the existence of a trade-off between progressivity and cost, with the models strongly agreeing that capital tax reductions are the most efficient but also the most regressive, whereas uniform lump-sum transfers to households are the most progressive but the least efficient. More generally, the models agree on the ranking of a recycling scheme across the progressivity and cost dimensions. While the magnitude of differences in welfare and consumption costs between schemes varies between models, there is broad agreement that capital tax reductions can greatly reduce the cost of CO_2_ taxation. For a representative tax starting at $25 per ton in 2020 and increasing at 5% a year to $63 in 2040, the models find average consumption losses of $21 to $173 per year and per capita if revenue is used to reduce capital taxes. If considering a longer time period, these often turn into net consumption gains.

We also investigate whether alternate revenue recycling schemes may offer a compromise in terms of equity and efficiency. For example, a scheme that reduces capital taxes but returns half of the revenue to households in lump-sum fashion eliminates the regressive nature of capital tax rebates. Such a scheme is found to be neutral or even slightly progressive in all models and comes at moderate additional cost ($99 to $250 average per capita annual consumption cost). We also find that the cost of protecting households in the lowest-income quintile is modest: two of the models have implemented hybrid policies which include additional transfers that leave the lowest income house-holds unaffected by the tax and find that these require only about 10% of the total revenue.

Finally, we briefly discuss disparities in impacts across sub-regions of the U.S. Although the models identify important differences in the welfare impacts of a CO_2_ tax across regions, there is here less agreement among models regarding their distribution, beyond the fact that the initially more energy-intensive regions face the greatest impacts.

This paper is organized as follows. Upon presenting the study design in Sec. 2, Sec. 3 discusses aggregate welfare outcomes. Section 4 then presents the distributional findings across models and tax revenue recycling schemes, which include lump-sum transfers and tax reductions, as well as scenarios meant to protect lower-income households. Section 5 describes the regional distribution of impacts. Throughout, we attempt to identify differences in modeling assumptions which may explain qualitative and quantitative disagreement across models.

## 2. Study Design

### 2.1. Scenario design

This study considers a set of scenarios that vary along two dimensions: (1) the CO_2_ price (tax) path and (2) the use of tax revenue. These CO_2_ price paths and the revenue recycling schemes align with those of the Energy Modeling Forum 32 (EMF32) model intercomparison project. Table 1 summarizes them and indicates which models produced output for which scenarios, with the models themselves discussed further below. Not all models reported all scenarios or all metrics so some figures in this paper will only display results from a subset of models.

Apart from a reference case with no CO_2_ price, the study considers scenarios with illustrative CO_2_ taxes starting at either $25 or $50 in 2020 increasing at either 1% or 5% annually. The taxes are economy-wide and apply to all CO_2_ emissions from fossil fuel combustion across all sectors, investment, and consumption. In our three core scenarios implemented by all models, the CO_2_ tax revenue, which is collected and pooled at the national level, is spent on a uniform lump-sum rebate to households (HH), on a capital income tax reduction (K), or on a labor income tax reduction (L). A subset of models implements a set of hybrid schemes. In one scheme, tax revenue is spent evenly between a capital tax reduction and a lump-sum rebate to households (K-HH). In another set of recycling schemes, revenue is used to keep the lowest-income quintile’s welfare unchanged while using the remainder either on capital tax reductions (TLQ-K), labor tax reductions (TLQ-L), or evenly between both labor and capital tax reductions (TLQ-L-K). A final recycling scheme keeps the lowest quintile’s welfare unchanged while ensuring progressivity across all income classes (P-TLQ-K).

### 2.2. Models

Of the models participating in the EMF 32 modeling exercise, four allow for the consideration of effects across income groups. These are DIEM (Ross, 2014a,b, 2018), USREP-ReEDS (Rausch and Mowers, 2014; Caron *et al.*, 2018), ADAGE (ADAGE-US) (Ross, 2009; Woollacott, 2018), and IGEM (Jorgenson *et al.*, 2012, 2013, 2018). These and a fifth model, NewERA (Tuladhar *et al.*, 2012), are able to consider the effects across U.S. regions.

Though all five models are based on general equilibrium modeling to obtain welfare impacts across income groups or regions including price changes as well as other general equilibrium effects, the models differ in many respects. All models except for ReEDS-USREP, which is a recursive-dynamic model, are full intertemporal optimization models with perfect foresight. Three — DIEM, USREP-ReEDS, and NewERA — include explicit representations of the electricity sector by coupling the economy-wide component of their models with detailed bottom-up electricity models. All have labor supply endogenously determined by a labor-leisure trade-off, but their treatment of capital supply varies.

In the remainder of this section, we discuss three characteristics that are of particular importance to the distributional insights from this paper, with an eye toward attempts to harmonize across models and limitations therein: (1) definition of and data source for quintiles; (2) how revenue is shared back under lump-sum transfers to households (HH); and (3) how labor and capital taxes are treated.

**Figure 1:**
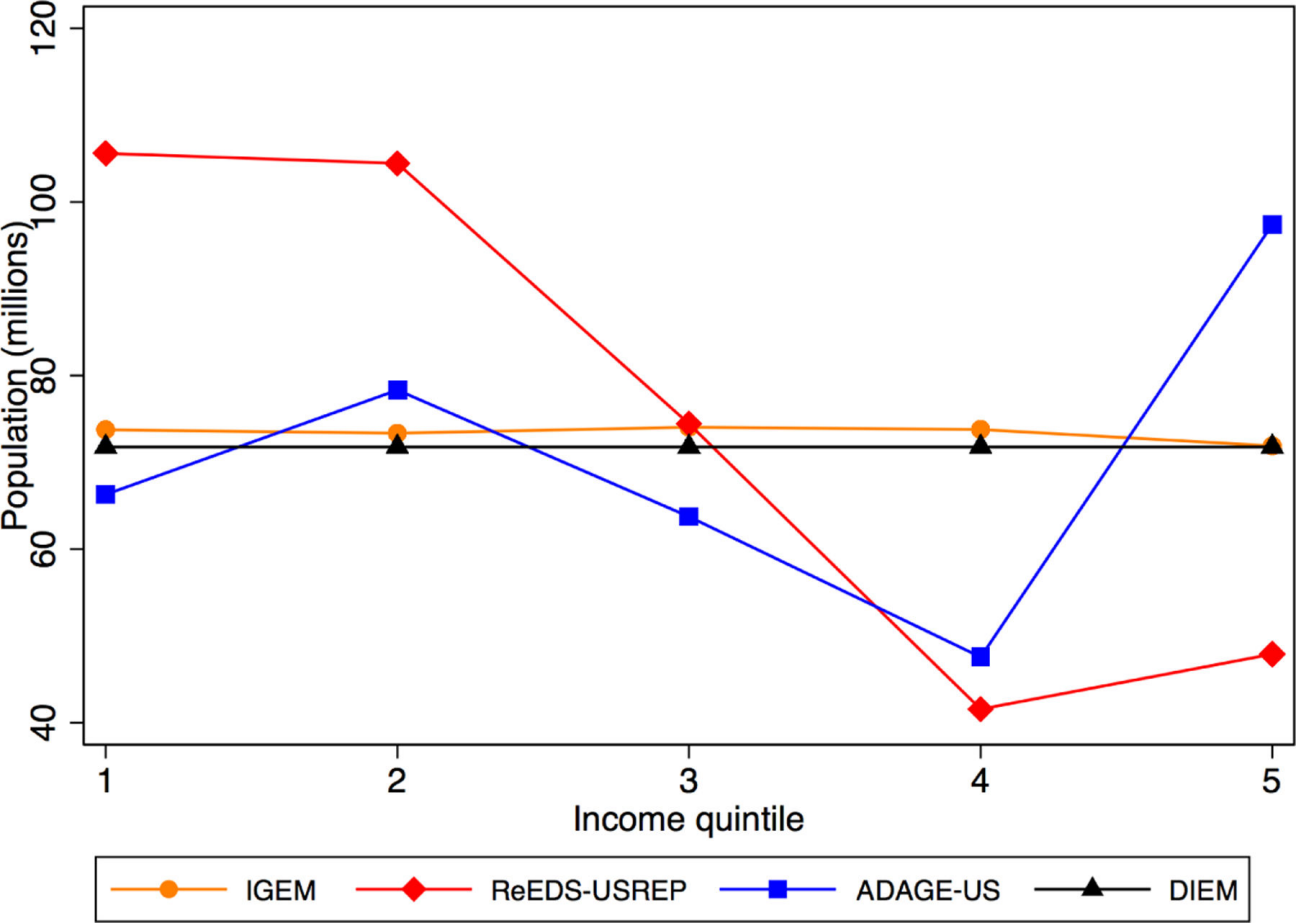
Average population over time across models. “1” represents the population quintile with the lowest income and “5” the largest income.

Distributional impacts across households of different income levels are harmonized and displayed in the results sections by quintile, sorted from the lowest to the highest-income quintile. Quintiles can be defined by population or households, and to the extent that household size varies by quintile, this choice could affect conclusions. ReEDS-USREP, ADAGE-US, and DIEM all use household income class data defined by annual income, based on the Consumer Expenditure Survey and described by IMPLAN social accounting matrices (IMPLAN, 2008; 2013), but differ in that the former two models define quintiles by household and the latter by population. IGEM represents 244 household types based on demographic and regional characteristics and assigns the persons within these to income quintiles that are as close to 20% each as possible; so, IGEM (like DIEM) defines quintiles by population. In all results, we standardize our results and present them by quintile as defined by population. For results presented as net present value (NPV), we use the present discounted value of population over time per quintile which varies substantially across models and income quintiles, as shown in Fig. 1.

Secondly, though care was taken to standardize scenarios across models, there are some differences in how the tax recycling scenarios are implemented in the models. Although all models pool the revenue nationally before distribution, they vary in how tax revenue is shared in the HH scenarios. ReEDS-USREP, ADAGE-US, and DIEM define uniform transfers on a per-household basis. IGEM, on the other hand, distributes tax revenue equally per capita.

Thirdly, though the capital (K) and labor tax (L) scenarios are equivalent across models in recycling all revenue back to reduce their respective taxes, the definition of these taxes varies as they consist of multiple taxes in the actual U.S. tax system. DIEM includes both corporate income and capital income taxes under capital taxes and labor income and payroll taxes under labor taxes. ReEDS-USREP includes these categories, but the tax reductions apply to marginal capital income and labor income taxes only. ReEDS-USREP and ADAGE-US compute marginal tax rates for each region, whereas IGEM and NewERA compute a single marginal tax rate for capital and income taxes. While the point of collection of the tax does in theory not affect the distribution of tax burdens, some differences may emerge from these definitional differences.

Finally, indexed transfer payments from the government to households are important sources of revenue for the households in the lowest quintile of the income distribution. All of the models in this study include such transfers, and baseline transfers are nominally exogenous in all cases. The indexing of transfers varies across models. In DIEM and ADAGE, they are indexed to the endogenous U.S. consumer price index (CPI). In USREP, they are indexed to a global CPI and in IGEM, where they are expressed nominally, they are not explicitly indexed and the real purchasing power of transfers and lump-sum rebates can potentially decline in the policy cases.

## 3. Aggregate Welfare Impacts

Before discussing distributional impacts across income quintiles and regions, we compare aggregate national welfare impacts across the models. We do so both over time and in present discounted value terms.

This paper focusses on the differential welfare impacts of a CO_2_ tax on households. We focus on two separate ways of reporting welfare impacts. The first reflects the tax’s impact on household consumption. While this is an incomplete measure of welfare, consumption is an important component of GDP that may be of interest to policy-makers. We report this metric in terms of $ of consumption loss per capita. The second metric we use is a measure of the change in welfare, or equivalent variation (EV). This reflects changes in household “full consumption”: consumer goods, consumer services, household capital services, and leisure — essentially all the elements entering the models’ household utility functions except for investment, which contributes to welfare in subsequent years. We report this metric in terms of a percentage change relative to household welfare in the no-tax reference. The distinction between consumption and EV matters most when comparing the L and K recycling schemes. Importantly, we note that welfare impacts discussed throughout the paper do not include the tax’s benefits from the reduced climate change externality.

We focus first on the three core recycling schemes, HH, K, L, and K-HH, which are simulated in most models. Emphasis will be put on the hybrid schemes in further sections.

### 3.1. Dynamics

**Figure 2:**
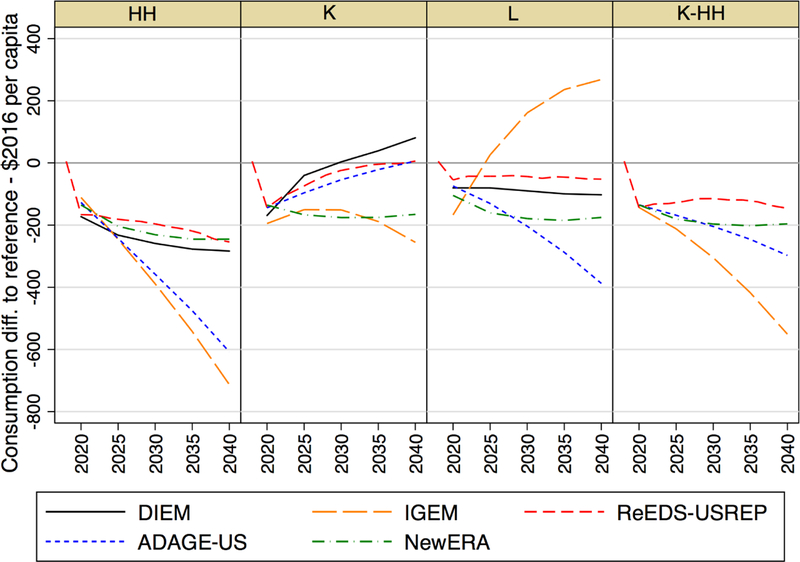
Time series of differences in average consumption per capita relative to the no-tax reference case, across revenue recycling schemes and models for the $25@5% CO_2_ price path.

**Figure A.1:**
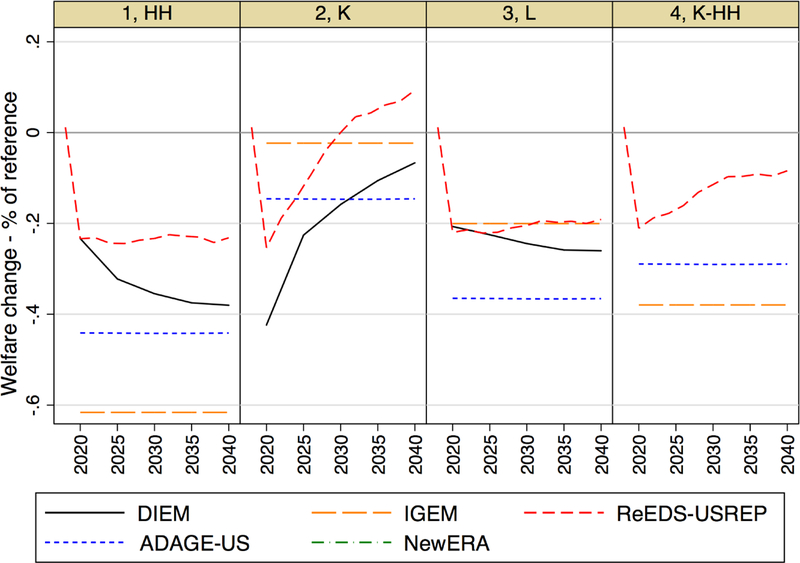
Time series of aggregate welfare change as percentage of the no-tax reference welfare, across revenue recycling schemes and models, $25@5% CO_2_ price path.

Figure 2 presents aggregate U.S. consumption loss in per capita terms for a $25 tax increasing at 5% a year across the models. Each panel represents a separate revenue recycling scheme: HH, household lump-sum rebates; K, a capital tax reduction; L, a labor tax reduction; and K-HH, half lump-sum rebates, half capital tax reduction. The equivalent graph for welfare change (expressed in percentage terms) is presented in Appendix A (Fig. A.1), but these results are less interesting as IGEM and ADAGE-US are completely intertemporal and relative welfare loss is therefore constant over time. Still, they reveal that constant per capita impacts in dollar terms actually reflect decreasing relative impacts in percentage terms.

Across all models, capital tax reductions (K) affect the rental price of capital services, promoting new saving, investment, and capital formation. This initially leads to suppressed consumption, as particularly notable in ReEDS-USREP, a dynamic-recursive model. Over time, however, the larger capital stock induced by the tax reduction raises incomes, consumption, and welfare. Consumption loss relative to the reference no-tax case thus decreases over time in most models, with households experiencing positive consumption impacts after about 2030 in DIEM and around 2040 in ADAGE and ReEDS-USREP. Note that capital income tax reductions also favor leisure over consumption as the latter is now relatively more expensive due to CO_2_ taxation. This effect is particular strong in IGEM: if considering changes in welfare instead (EV), this model also finds capital income tax rebates to be least costly.

Reducing labor taxes (L) promotes consumption and works through real-wage incentives that compensate for the effects of carbon pricing and is the next most favorable recycling scheme in welfare terms, as seen in Fig. A.1, but this occurs at the expense of saving, investment, and capital formation. Labor tax reductions actually lead to increased consumption in IGEM.

Lump-sum redistribution of CO_2_ tax revenues to households (HH) is the least favorable recycling option. It incentivizes neither capital nor labor. Consequently, the declines in overall social consumption and welfare are the greatest among the three schemes. These effects are reduced when dividing the income between lump-sum rebates and capital income tax reductions (K-HH).

### 3.2. Net present values

**Figure 3:**
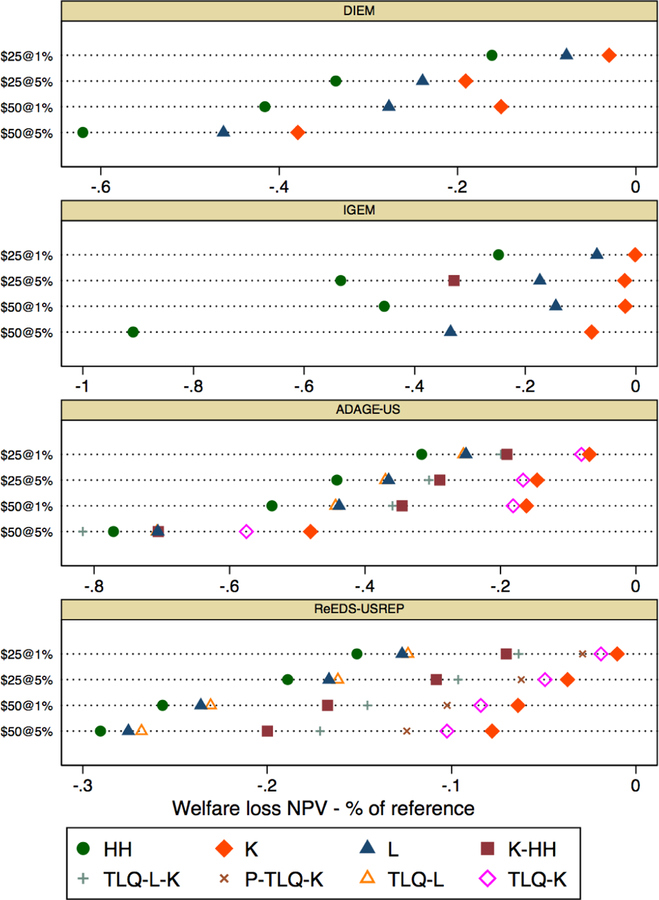
Percentage change in the 2020–2040 NPV of welfare, for different CO_2_ price paths and revenue recycling schemes across models.^2^ Note that the axis scaling differs by model.

**Figure 4:**
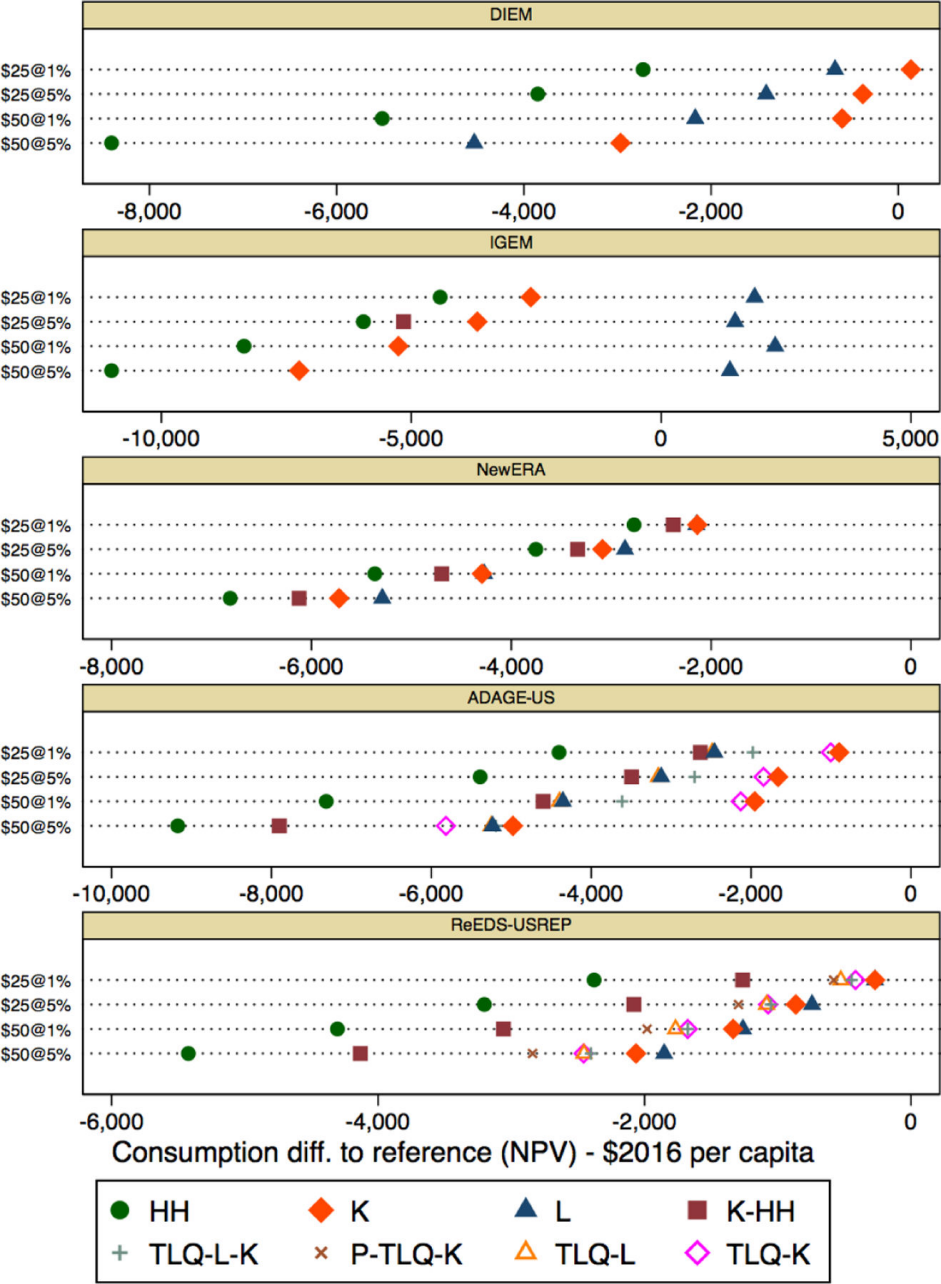
Difference in the NPV of consumption relative to reference case in $ per capita, 2020–2040 NPV, for different tax rates and revenue recycling schemes across models.

We now consider aggregate welfare and consumption impacts over the entire period from 2020 to 2040 in present discounted value terms. We compute the NPV (with linear interpolation between years) using a 3% discount rate. Results are presented graphically in Fig. 3 for changes in welfare (% change in the NPV), whereas consumption changes are shown in Fig. 4. The numbers underlying these graphs, i.e., welfare and consumption changes for all combinations of models, revenue recycling scheme, and CO_2_ price path, are given in tabular format in Table A.2.^1^

Figure 3 presents the percent change in the NPV of welfare, with models represented in each panel, recycling schemes represented by color, and the CO2 price paths on the vertical axis. Though there is a large difference in the magnitude of impacts between models, this figure reveals a very clear agreement regarding the ordering of welfare costs, with lump-sum transfers to households (HH) being the most costly and capital tax reductions (K) being the most efficient. The robustness of this result across models and CO2 price paths is in line with the existing literature on the efficiency of capital tax reductions. Notably, this result holds both for recursive-dynamic models (ReEDS-USREP) and intertemporally optimized models (the others).

The models also agree that labor income tax rebates (L) have welfare costs which lie in between those of K and HH, and most agree about the overall ranking of the “hybrid” schemes (K-HH and the TLQ scenarios).

However, there is less agreement on relative differences between recycling schemes. The quantitative differences in the average welfare costs across models and the sometimes limited agreement on relative differences between recycling schemes can be explained by differences in the assumptions underlying the models. For instance, the gaps between recycling schemes are largest in IGEM likely due to differences in the ways the various tax mechanisms affect model outcomes (e.g., capital prices versus capital incomes, real wage incentives, and labor supply responses). Also, in IGEM, leisure represents a larger share of full consumption than in the other models, as it trades off directly against consumer goods, services, and capital, instead of an aggregate of these in a separate nest. Finally, while welfare costs generally increase with tax stringency in all models, the differences between the $25@5% and $50@1% paths is not robust, so models do not agree on the optimal price path. The simulation horizon in IGEM is 2015–2130 so the post-2050 period matters in terms of abatement and welfare. Thus, for IGEM, the $25@5% price path is more costly than the $50@1% trajectory. Other differences in the average cost of taxation are due to differences in the set of available abatement technologies. The ReEDS-USREP model, for instance, allows for rapid re-dispatch of electricity generation to lower CO_2_-intensity natural gas generation in the short term and for relatively large renewable energy shares in the medium term. This leads, overall, to relatively modest welfare costs.

Figure 4 presents results in the same format as Fig. 3, but for consumption, which we express as the change in NPV per capita consumption in $ terms.

Figure 4 reveals much more agreement between the models for the magnitudes of consumption loss under lump-sum transfers to households (HH). Also, as discussed in the time-series results, the impacts of the labor income tax reduction scheme by comparison are smaller, as labor tax reductions benefit consumption. Moreover, the reduction in leisure is not reflected here as it is in welfare. For consumption, CO_2_ taxation with labor tax reductions is superior to capital tax reductions in IGEM under all tax parings and in ReEDS-USREP for the higher tax paths.

## 4. Distributional Impacts by Income Quintile

We now focus on the differentiated effects of CO_2_ taxation on households of different income levels. All results are summarized here by income quintile. For a given CO_2_ price path, the choice of revenue recycling scheme has a significant impact on the distribution of welfare outcomes across household quintiles. The benchmark level of welfare in each quintile varies across models, so we present relative measures such as percent changes or dollars per capita to allow for better inter-model comparison.

### 4.1. Dynamics

**Figure 5:**
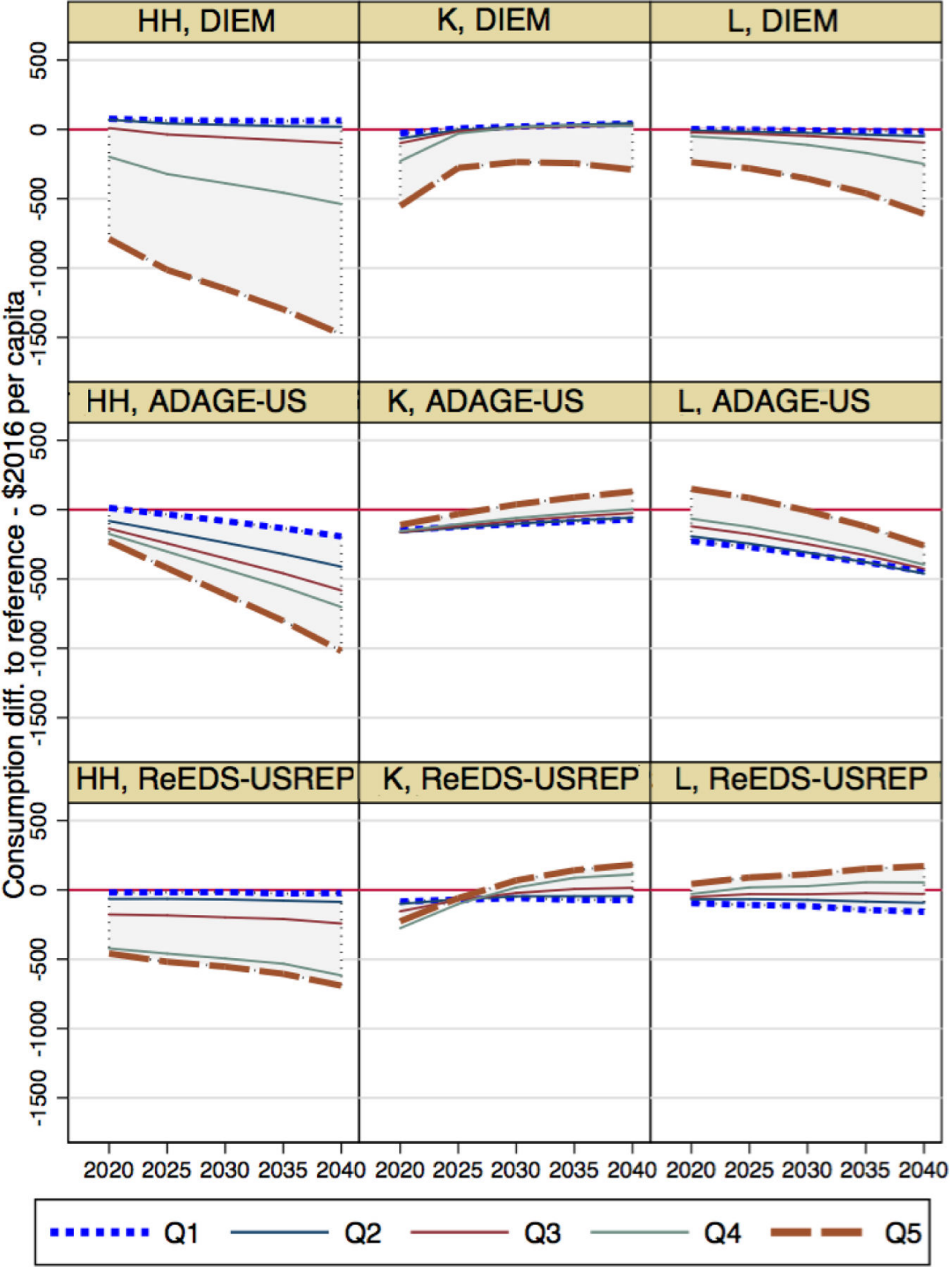
Time series of consumption loss per capita for each income quintile relative to the reference case across revenue recycling schemes and models for the $25@5% CO2 price path. “Q1” represents the lowest-income quintile and “Q5” the highest-income quintile.

We first consider how per-capita consumption loss evolves over time across income quintiles in Fig. 5 for the $25@5% price path. Rows represent results, per model, for the three core revenue recycling schemes. The models agree that a uniform lump-sum distribution to households is progressive, with the lowest-income quintile either facing a modest consumption loss or a very slight gain. The standard deviation of these losses increases over time in proportion to the increasing tax stringency.

For capital and labor tax reductions, however, there is less agreement. Still, the capital tax reduction (K) is found to be regressive in two of the three models: ADAGE and ReEDS-USREP, as well as in IGEM (not shown as it solves for full welfare, not consumption, at the household level). In ReEDS-USREP, it switches from progressive in the first couple of years to regressive in the long term when measured in terms of consumption changes. This change does not occur when considering welfare (not shown). Capital income tax reductions are progressive in DIEM. In all models, they lead to less variability between income quintiles than under HH or L.

A labor tax reduction (L) is regressive in two of the models and progressive in DIEM. IGEM finds it to be mostly neutral in terms of full welfare. In addition, the standard deviation of per-capita consumption changes increases for two of the models, but decreases for ADAGE-US.

Although DIEM thus stands out in terms of the progressivity of K and L, it is in agreement with other models in that both K and L are substantially less progressive than HH.

### 4.2. NPV summaries, by income quintile

We now turn to distributional impacts in NPV terms. Quintiles differ in terms of the size of their reference consumption and welfare. For comparability across quintiles and to convey quintile-level welfare impacts, we have focused our results on percent changes.

**Figure 6:**
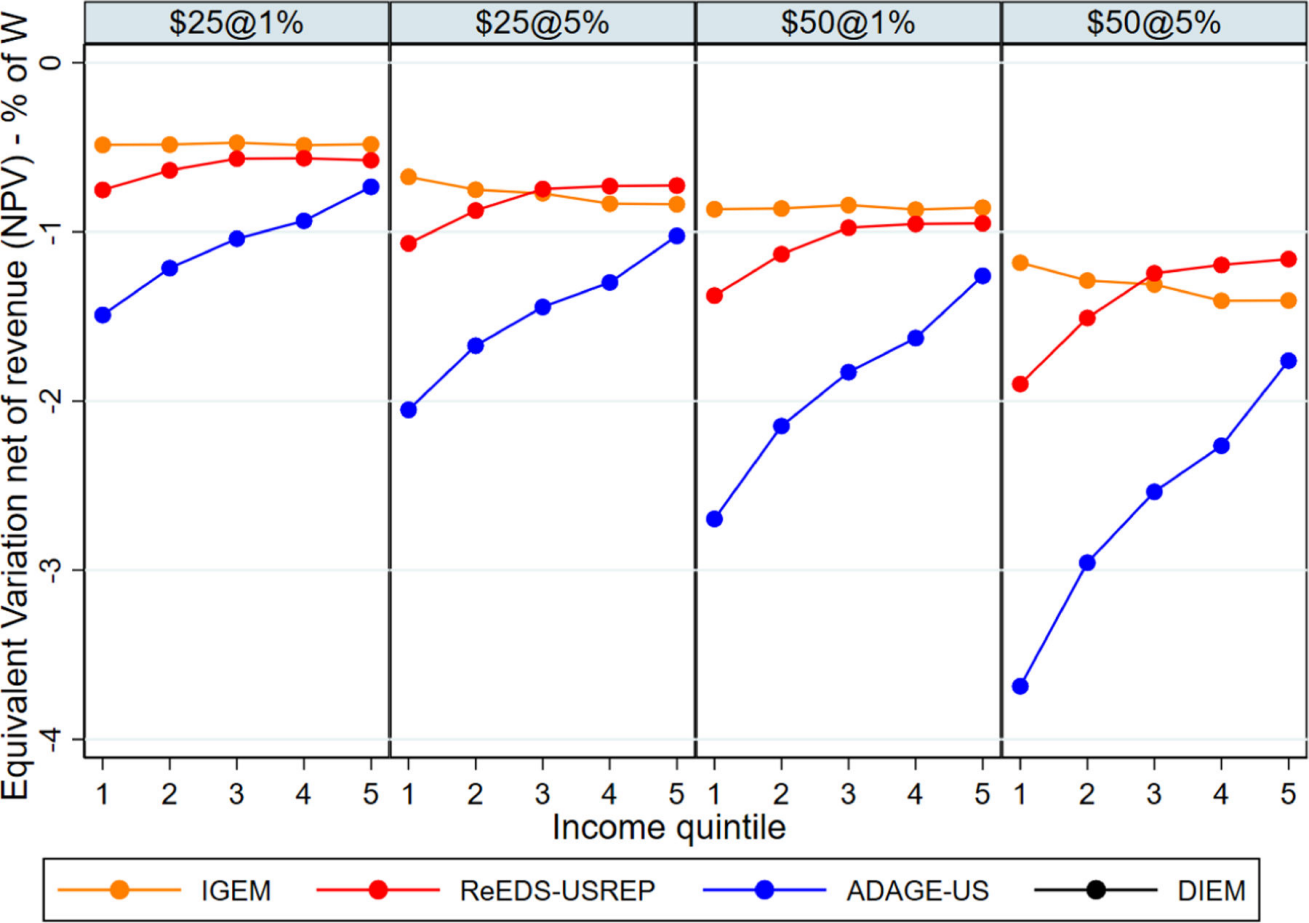
Percent welfare change (NPV), relative to the no-tax reference, net of recycled revenue.

We first examine the gross cost of the carbon policies on households, that is, the impact the policy would have had without any carbon revenue recycled. By first examining the gross policy cost, we establish a baseline impact against which we can compare the re-distributional impact of different recycling methods. To do so, Fig. 6 shows the percentage welfare impact on households by income quintile under the lump-sum scenario (HH), but with the lump-sum revenue subtracted from households’ welfare. The exact costs of abatement, gross of recycling benefits, are impossible to obtain in general equilibrium models such as those under consideration here. Still, the gross-of-lump-sum recycling revenue measure provides a reasonable approximation in as much as lump-sum transfers do not substantially reduce or increase pre-existing distortions to the economy. With this measure, we approximate the gross abatement cost that would occur if the revenue was not recycled at all. At the time of writing, DIEM did not report the revenue recycled so this exercise could not be undertaken for that model.

The results of ReEDS-USREP and ADAGE-US suggest that the CO_2_ tax alone (i.e., without any form of revenue recycling) has a regressive impact on households, whereas IGEM model results are largely neutral across quintiles, with some suggestion of progressivity for the highest tax rates. ReEDS-USREP and ADAGE-US results also suggest that the regressivity of the policy may be exacerbated by higher rates, as evidenced by their steepening slopes moving from left to right in Fig. 6.

**Figure 7:**
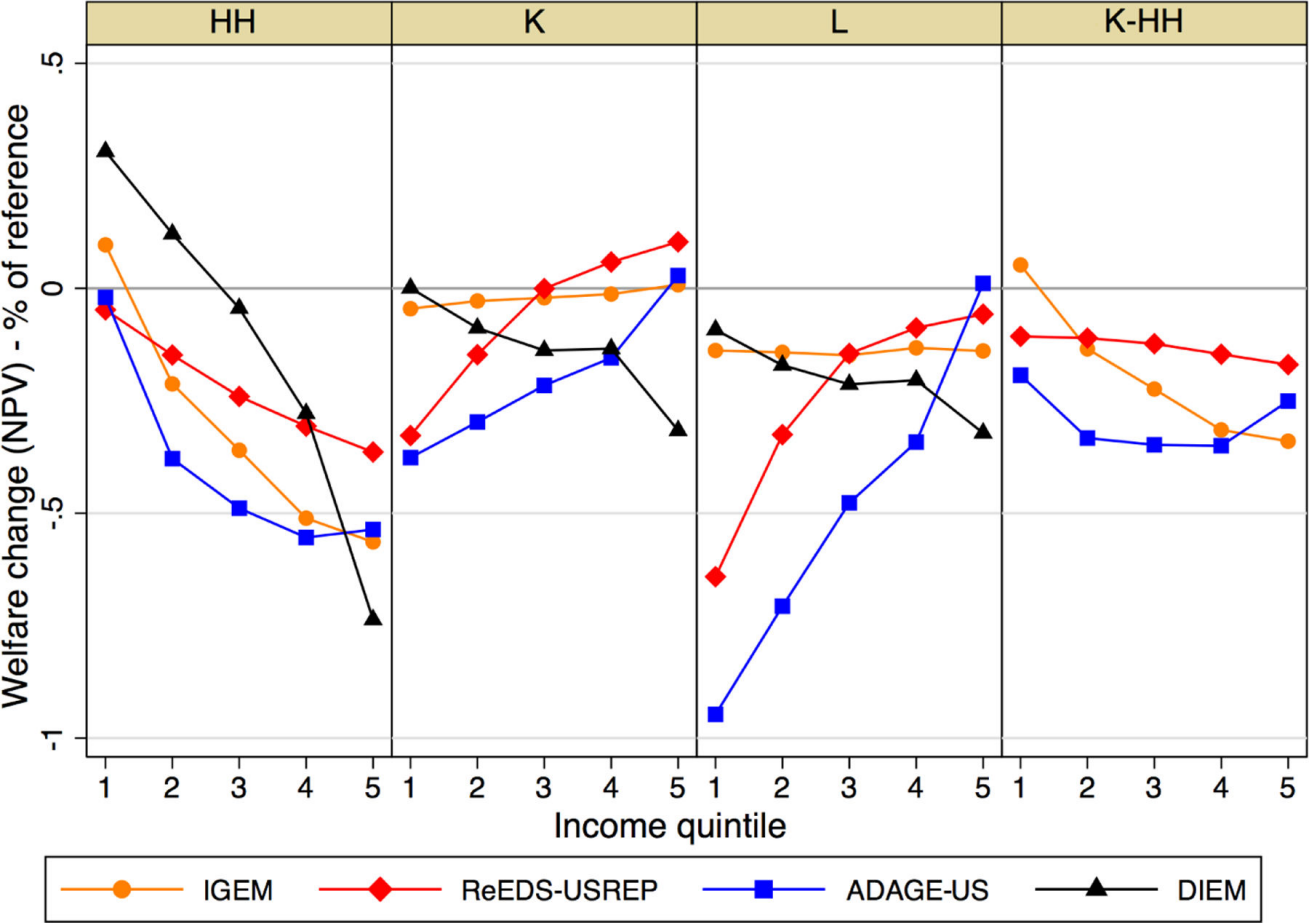
Percent change in NPV welfare (relative to reference welfare) by recycling scheme for the $25 tax rate rising at 5% CO_2_ price path.

Next, we examine how different recycling schemes alter the distribution of the gross impacts shown in Fig. 6. Figure 7^3^ shows the results across different revenue recycling schemes at a $25 tax rate rising at 5%. There is inter-model agreement that a simple lump-sum approach is progressive, in some cases even making lower-income house-holds better off under the policy. Households with higher levels of pre-policy welfare are proportionally more affected by increased expenditures and lower factor returns incomes and profit relatively less from the fixed, lump-sum value of recycled revenue.

ReEDS-USREP and ADAGE-US show both capital and labor tax rebates as having a clearly regressive impact on households. In the case of capital tax rebates, three of the four models report highest-income households as better off under the policy. DIEM results show progressive impacts in the capital and labor tax scenarios, though significantly less so than in the lump-sum scenario. Higher-income households are better off than under HH in all models.

The distributional effect of direct (non-CO_2_) government transfers to low-income households may partially explain differences in impacts for the lowest quintile across scenarios. They likely push the CO_2_ taxes toward progressivity, as low-income households derive smaller shares of their total income from labor/capital. However, the overall regressivity of the K and L schemes in three of the models suggests that their role is limited. The differential treatment of transfer indexing may explain differences on impacts for low income households such as those observed from DIEM: the indexing assumed in that model makes low-income households benefit from the economic growth stimulated by the tax rebates.

Finally, the mixed capital and lump-sum recycling scheme K-HH captures the efficiency gains of reduced capital taxes (that help offset gross policy costs) while largely neutralizing the regressivity of such tax reductions. ReEDS-USREP and ADAGE-US show a mostly neutral to slightly progressive impact across households under K-HH, whereas IGEM results more clearly exhibit some of the progressivity seen in the lump-sum only scenario.

**Figure 8:**
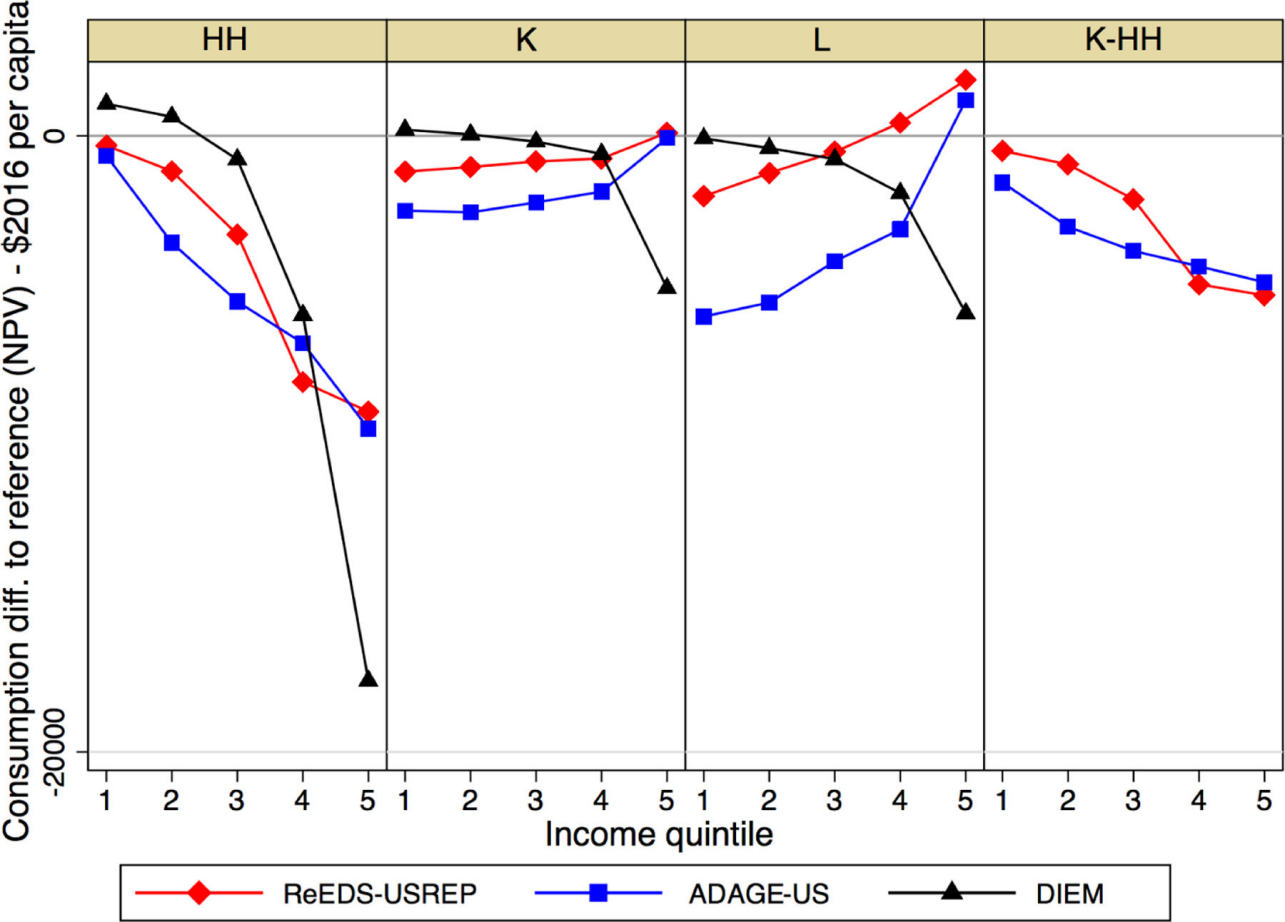
NPV consumption loss per capita across revenue recycling schemes for a $25@5% tax. *Note*: Consumption is not split out by income quintile in IGEM and thus cannot be displayed here.

For a $25 tax rate rising at 5%, as plotted in Fig. 8, per capita changes in consumption range from gains to a few hundred dollars of losses per person, depending on the scenario. Although the shape of the distribution of impacts varies, the overall patterns are similar to those found in Fig. 7 for percent welfare changes. Lump-sum recycling is progressive, with lower-income households experiencing per-capita increases in consumption in DIEM. Interestingly, L and K, while still more regressive than HH, seem to offer less of an advantage to high income households in all models. In ReEDS-USREP and ADAGE, the curves are flatter. For DIEM, the highest-income quintile is considerably more affected than the other quintiles. The K-HH revenue recycling scheme would be perceived as progressive if measured using consumption instead of welfare.

### 4.3. Measures of inequality

The results so far suggest that revenue recycling schemes vary in terms of both efficiency (reflected by differences in aggregate costs) and equity (reflected by differences in the distribution of impacts). To better summarize differences between schemes, we construct two measures of the inequality in impacts and evaluate them relative to the aggregate cost of the policy. By this comparison, we can assess the extent to which a trade-off between economic efficiency and equity is apparent in the results.

**Figure 9:**
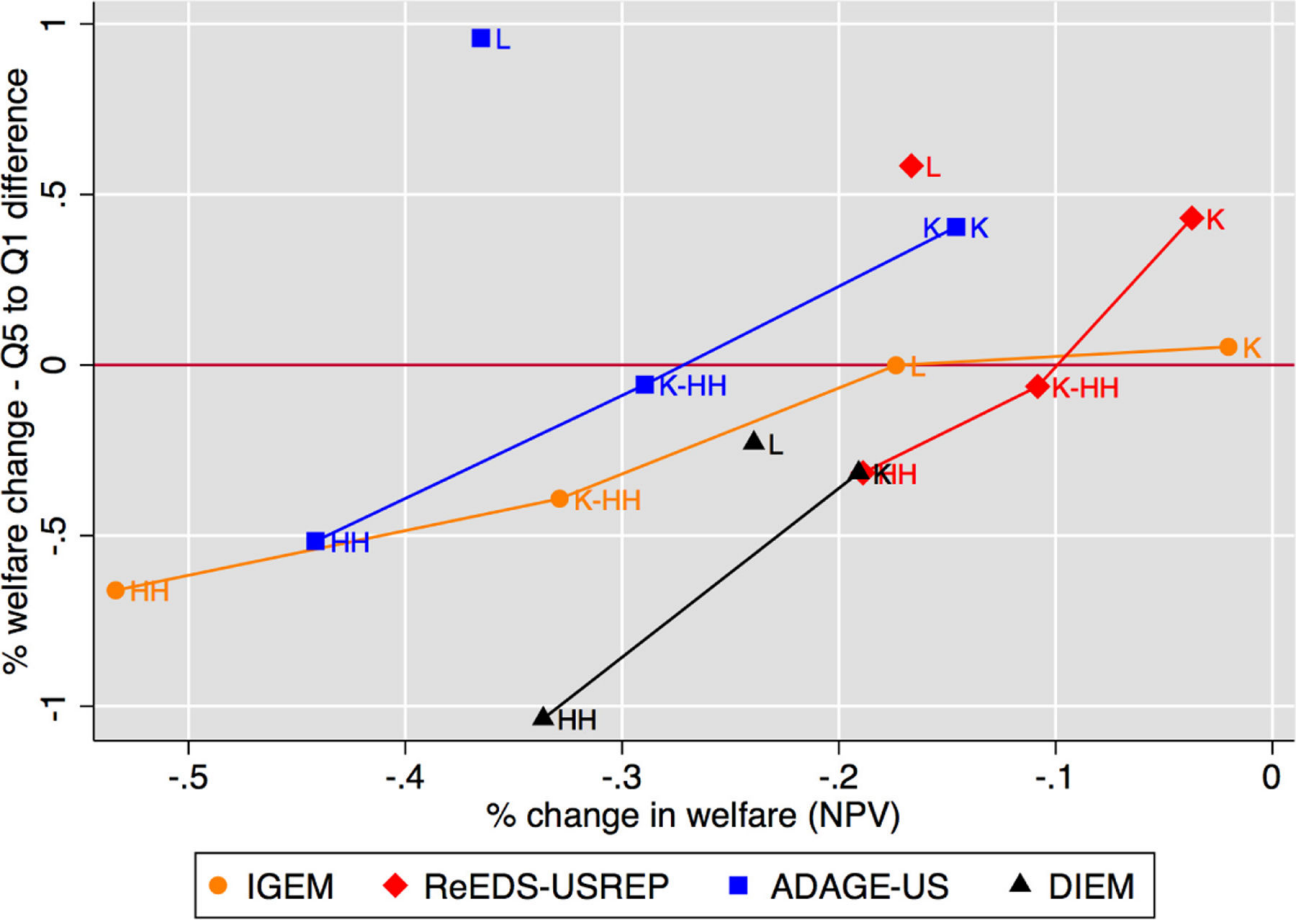
The trade-off between progressivity and aggregate welfare costs for a $25 at 5% tax, with progressivity measured by the difference between impacts on quintile 5 and quintile 1. Schemes toward the right are less costly; schemes toward the bottom are more progressive.

Our first measure of progressivity simply reflects the difference between the percentage welfare change incurred by the fifth (highest-income) and the first (lowest-income) quintiles. Figure 9 displays this metric on the vertical axis with aggregate welfare cost on the horizontal axis and thus identifies recycling schemes that are more progressive (those that are to the bottom of the graph) and least costly (those that are toward the right).

**Figure 10:**
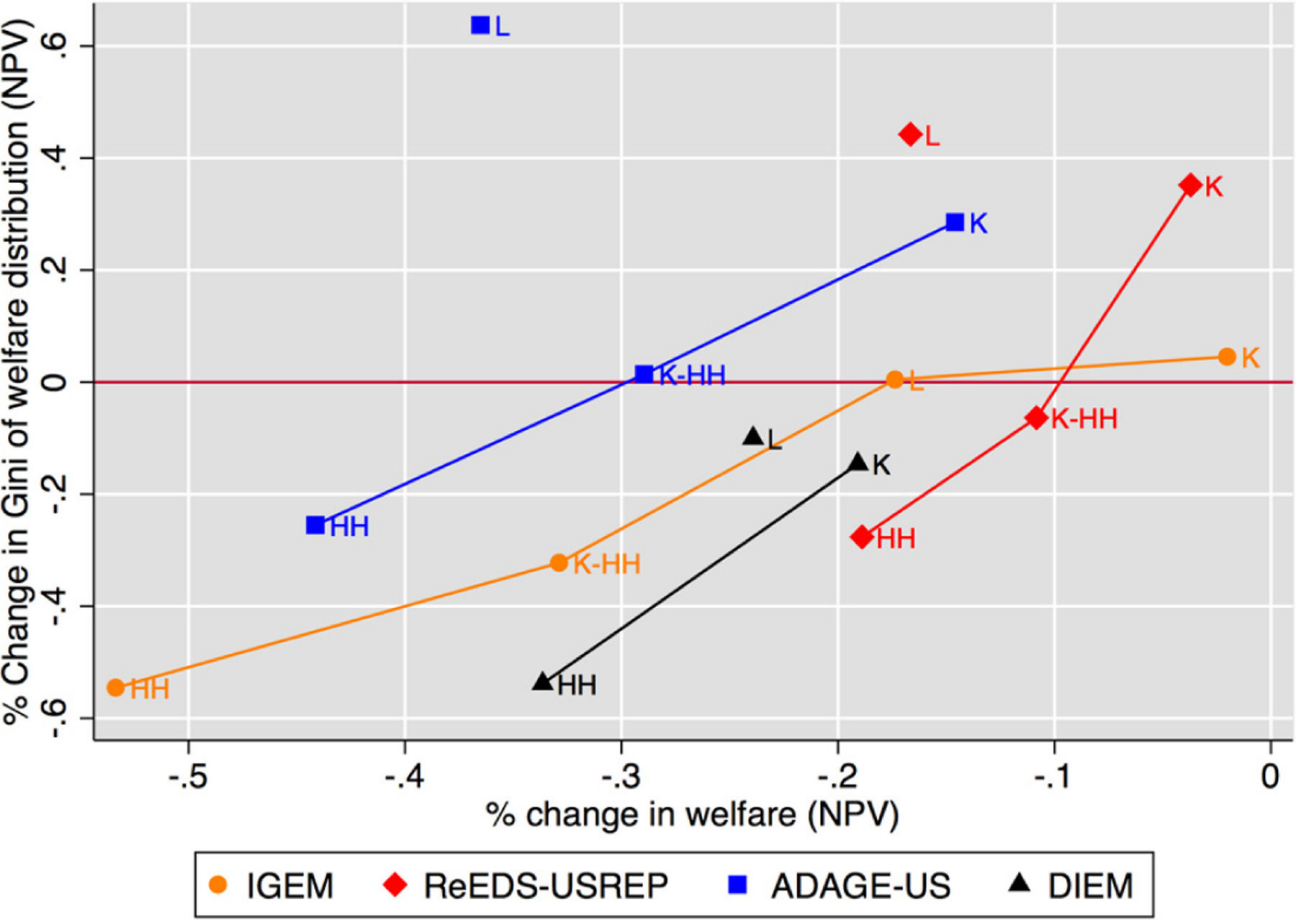
The trade-off between progressivity and aggregate welfare costs for a $25 at 5% tax, with progressivity measured by the tax-driven change in the Gini coefficient of the welfare distribution. Schemes toward the right are less costly; schemes toward the bottom are more progressive.

Our second measure of progressivity relies on the Gini coefficient, a common measure of inequality. In particular, it represents the percentage change in the Gini coefficient of the welfare distribution caused by the CO_2_ tax, relative to the Gini of welfare distribution in the no-tax reference. A positive value implies that the policy has increased the welfare distribution’s inequality and that the policy is thus regressive. Figure 10 displays this metric on the vertical axis^4^ with aggregate welfare cost on the horizontal axis and again identifies recycling schemes which are more progressive and least costly (those that are toward the bottom right of the graph).

Both Figs. 9 and 10 reveal a clear trade-off between efficiency and equity: recycling schemes that are more efficient (less costly) are on average more regressive. All models agree on this. To make this trade-off clearer, we have connected the schemes that are not dominated by another scheme in either dimension. The model lines in the figure all exhibit a positive slope, indicating the presence of an equity–efficiency tradeoff. For all models, the lump-sum and capital tax rebate scenarios set the positive extremes of progressivity and economic efficiency, respectively.

Apart for IGEM, lines for each model connect all but the labor tax reduction scenario, which is inferior in terms of both efficiency and equity relative to capital tax reductions. The exception is IGEM which finds L to be slightly less regressive than K. However, for a given level of aggregate welfare loss, the labor tax recycling scenario imposes much more regressivity than would be expected based on any mix of the lump-sum and capital scenarios. Therefore, there is agreement across models that a combination of capital tax and lump-sum rebates would be a preferred means for targeting a particular mix of equity and economic efficiency.

The K-HH recycling scheme (not modeled by DIEM) generally lies somewhere between HH and K, being dominated by neither and illustrating the fact that different combinations of equity and efficiency are attainable through combinations of tax reductions and direct transfers.

**Figure A.5:**
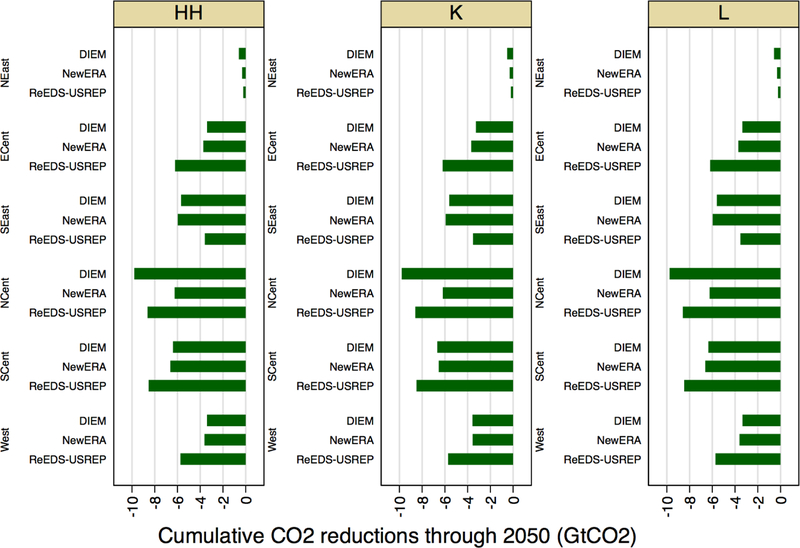
Cumulative CO_2_ reductions through 2050 (GtCO_2_) by region for a $25@5% tax.

The similarities between Figs. 9 and 10 show that these results are robust to the way the inequality in impacts is measured. Results are also largely robust to tax stringency, as can be seen in Fig. A.5 of Appendix A, with the exception of L in ADAGE which is considerably more progressive under the stringent $50 at 5% price path. It is even more costly, and remains dominated, though this time by HH and not K. The figure also indicates that the range of efficiency costs and impact inequality increases with higher tax rates.

### 4.4. Impacts on low income households

Results so far indicate that while the lowest-income quintile of households would be unaffected or even profit from CO_2_ taxation under the lump-sum rebate scheme, it would be negatively impacted under capital income tax rebates, especially when costs are measured relative to reference welfare. It is the most affected income group in all models but DIEM. At the same time, results unambiguously reveal the efficiency of capital income tax rebates.

To the extent policy-makers take interest in mitigating the regressivity of the CO_2_ tax’s gross impacts, it is worthwhile assessing how much of the efficiency gains provided by capital income tax reductions must be sacrificed to limit impacts on the poorest households. To do so, ReEDS-USREP and ADAGE-US simulated a set of revenue recycling schemes. These are specific to this paper and not included in the EMF 32 study. We denote these scenarios by “TLQ”, reflecting the fact that the lowest-income quintile’s welfare is left unaffected by the tax through the introduction of endogenously determined lump-sum transfers to these households, with the remaining revenue recycled through capital and/or labor tax rebates. In ReEDS-USREP (a recursive-dynamic model), the constraint is imposed in each period, whereas in ADAGE (a perfect foresight model), the NPV of welfare is held constant over the model horizon.

Finally, ReEDS-USREP also simulates a scenario in which the revenue is used to provide additional transfers to all but the richest income quintile so as to insure the overall progressivity of the tax across all quintiles in relative terms (P-TLQ). The size of these transfers is determined such that the welfare impact of each household quintile, in percentages, is not any larger than the next richest quintile.

Figure 12 (and corresponding numbers in Table A.3) presents the reduction in welfare costs achievable by various recycling schemes, relative to proportional lump-sum transfers (HH), the costliest scheme. First, it reveals once again the substantial savings that capital tax rebates can generate, in terms of aggregate welfare: more than 66% lower welfare cost in both ReEDS-USREP and ADAGE-US for the $25@5% price path. This percentage would be even larger if a longer time horizon was considered, as the efficiency gains of capital tax rebates increase in time.

Relative to this cost-saving, the welfare cost of setting some of the revenue aside for lump-sum transfers to lowest-income quintile households (the “TLQ” schemes) is low, especially if all of the remaining revenue is used to lower capital taxes, as in TLQ-K. In this case, the results of ReEDS-USREP indicate that lowest income households can be compensated by sacrificing only 6.5% points of the efficiency gains of K (with savings relative to HH of 73.9% instead of 80.4%), whereas ADAGE-US results indicate that they can be compensated by sacrificing only 4.7% points of K’s efficiency gains (with savings relative to HH of 62.3% instead of 67.0%). These results are mostly consistent across carbon tax levels, although the relative cost of compensating the lowest-income quintile increases with the most stringent price path.

**Figure 11:**
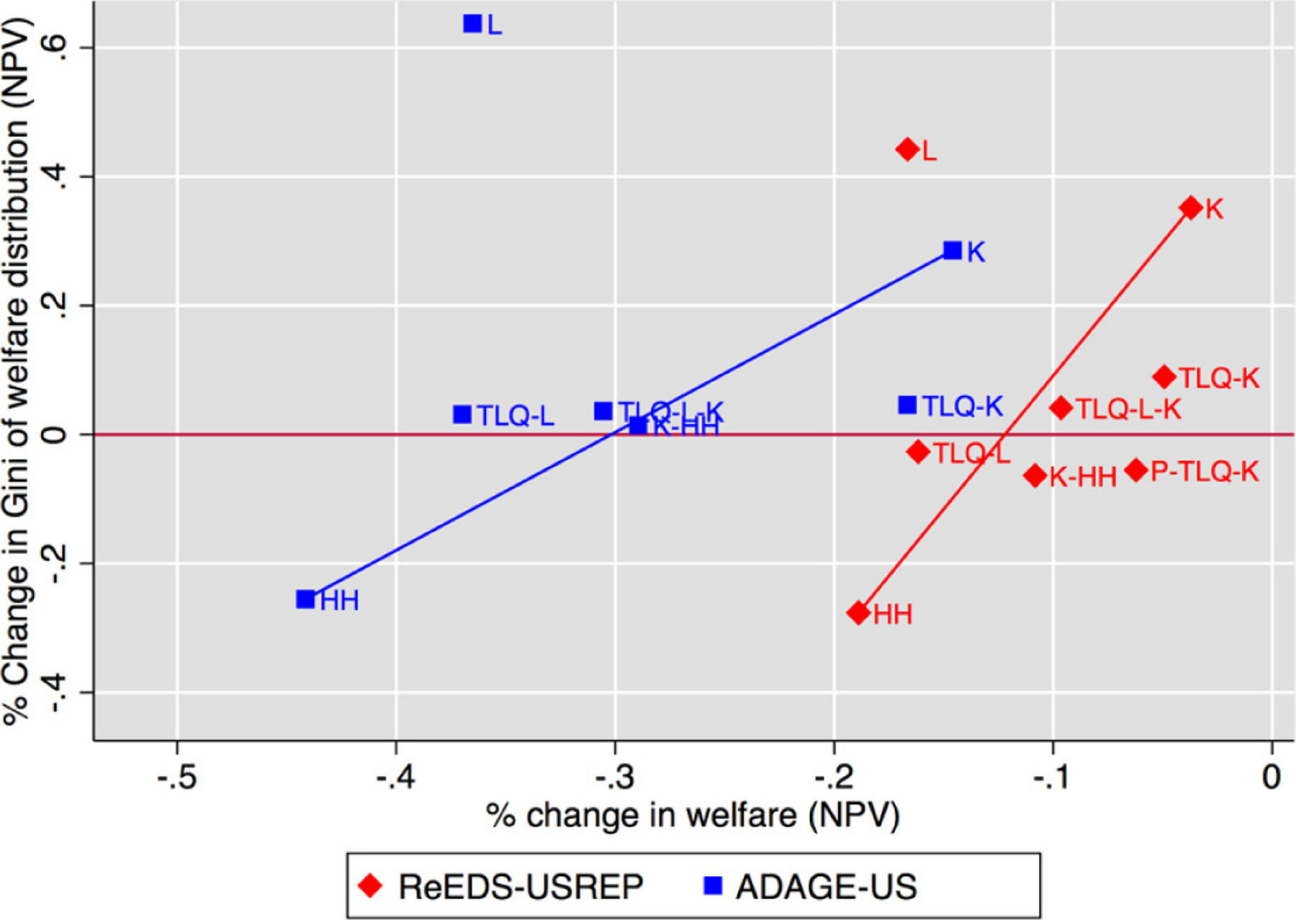
The trade-off between progressivity and aggregate welfare costs for $25 at 5% tax including hybrid TLQ and P-TLQ schemes. Schemes toward the right are less costly; schemes toward the bottom are more progressive.

**Figure 12:**
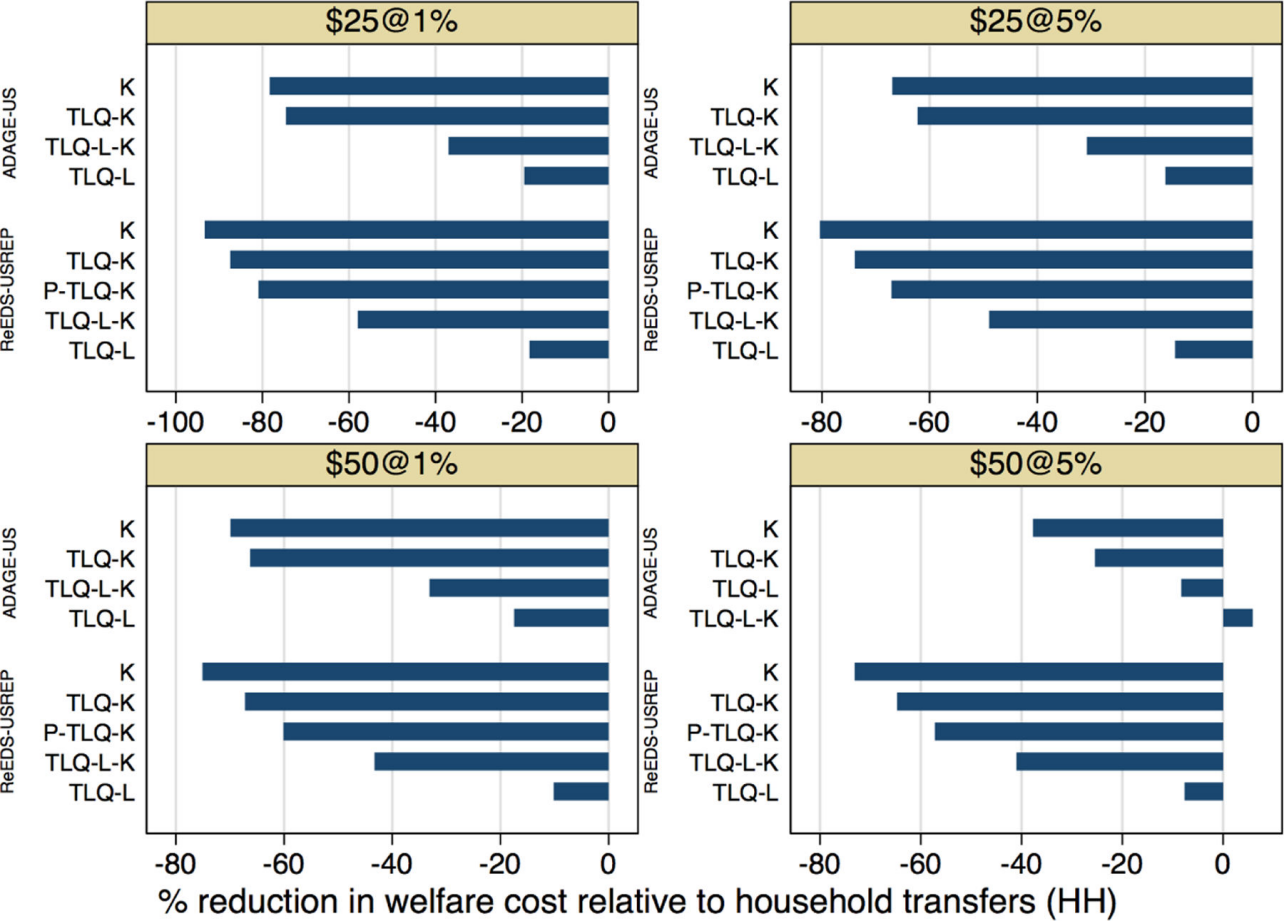
Aggregate welfare cost of neutralizing economic impacts on lowest-quintile households: percentage reduction in welfare cost afforded by each scheme relative to HH.

Overall, these two models do not suggest that there is any advantage to using some (TLQ-L-K) or all (TLQ-L) of the revenue to reduce labor taxes. This result comes from labor income tax reductions not substantially helping the lowest-income households, given that they derive a substantial share of their income from fixed government transfers. Figure 11, which plots these policies on the same efficiency–equity space as was done on Fig. 10, shows that the TLQ-L-K and TLQ-L schemes are inferior to other schemes in both models.

Figure 11 makes it clear that a number of schemes dominate points on the trade-off line between HH and K. It is thus possible to improve on this trade-off with hybrid schemes.

In addition, the ReEDS-USREP model finds that adding an overall progressivity requirement on the policy (TLQ with additional rebates to all households to insure progressivity with capital tax recycling) in P-TLQ-K was also possible at low aggregate efficiency costs over and above the same policy without the progressivity requirement.

**Figure 13:**
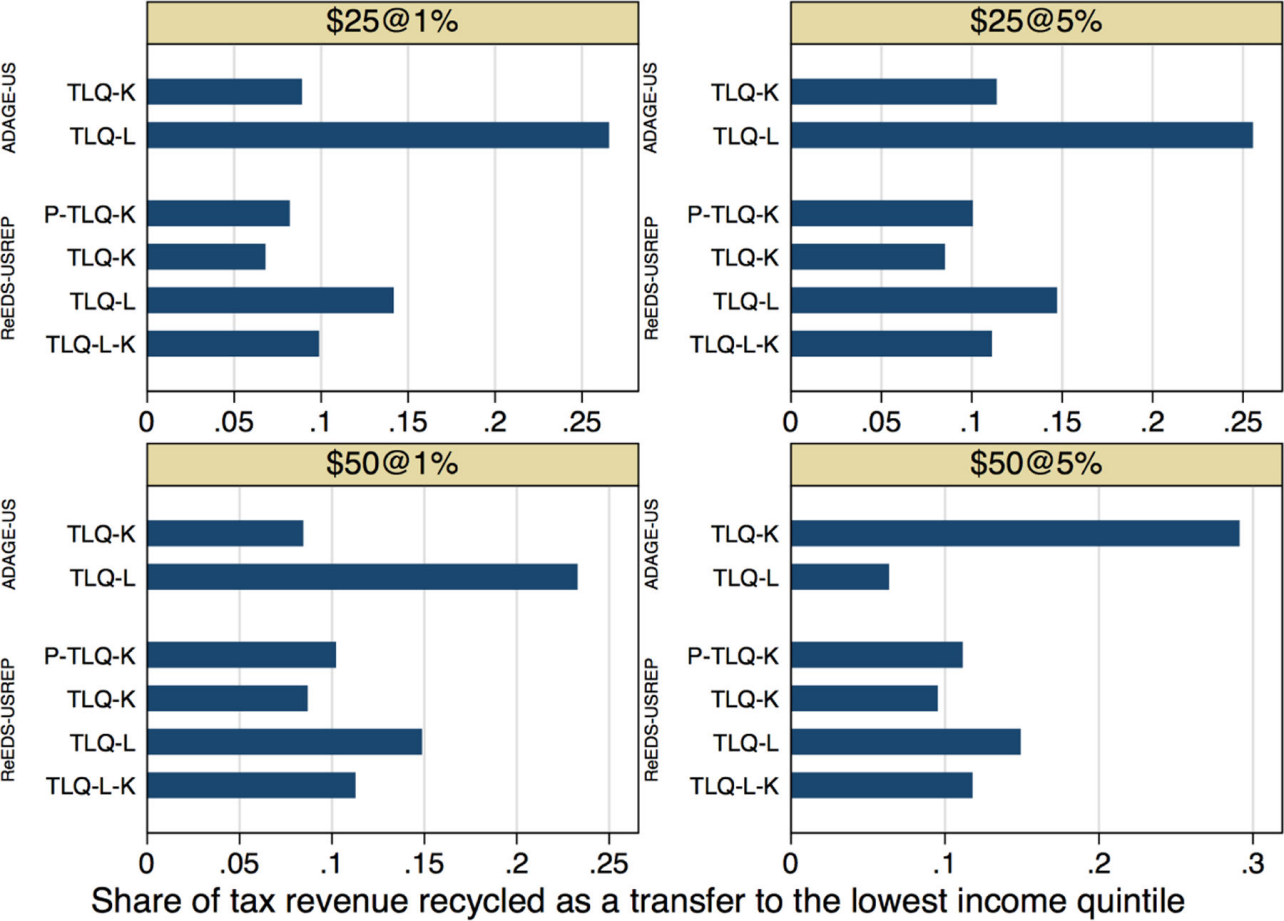
Revenue requirements of neutralizing welfare impacts on the lowest-income quintile of households (share of total tax revenue available for recycling).

Finally, Fig. 13 (also Table A.4) helps clarify why the aggregate welfare costs of transfers to compensate low income households or insure progressivity are modest by showing the share of total CO2 tax revenue required to neutralize the policy for lowest-income quintile households. Roughly 10% of revenue is needed to neutralize the policy for these households under a capital tax rebate.

Overall, the aggregate welfare cost of neutralizing the policy for lowest-income quintile households while recycling the remainder to capital tax rebates appears modest in both models. The results here suggest that equity goals can be achieved via targeted lump-sum transfers at a modest cost to aggregate welfare.

## 5. Regional Impacts

This section presents the distribution of impacts across regions. Although all four models discussed earlier as well as NewERA explicitly describe sub-regions of the U.S., we focus results on the three whose regional definitions align: DIEM, USREP, and NewERA. These regions are broadly based on Census regions and are defined in Table A.1 of Appendix A.

Throughout this section, we present results for the scenarios with a $25/ton CO2 tax increasing at 5% a year. Though the results are quantitatively different across different CO2 price trajectories, the qualitative conclusions are similar.

**Figure 14:**
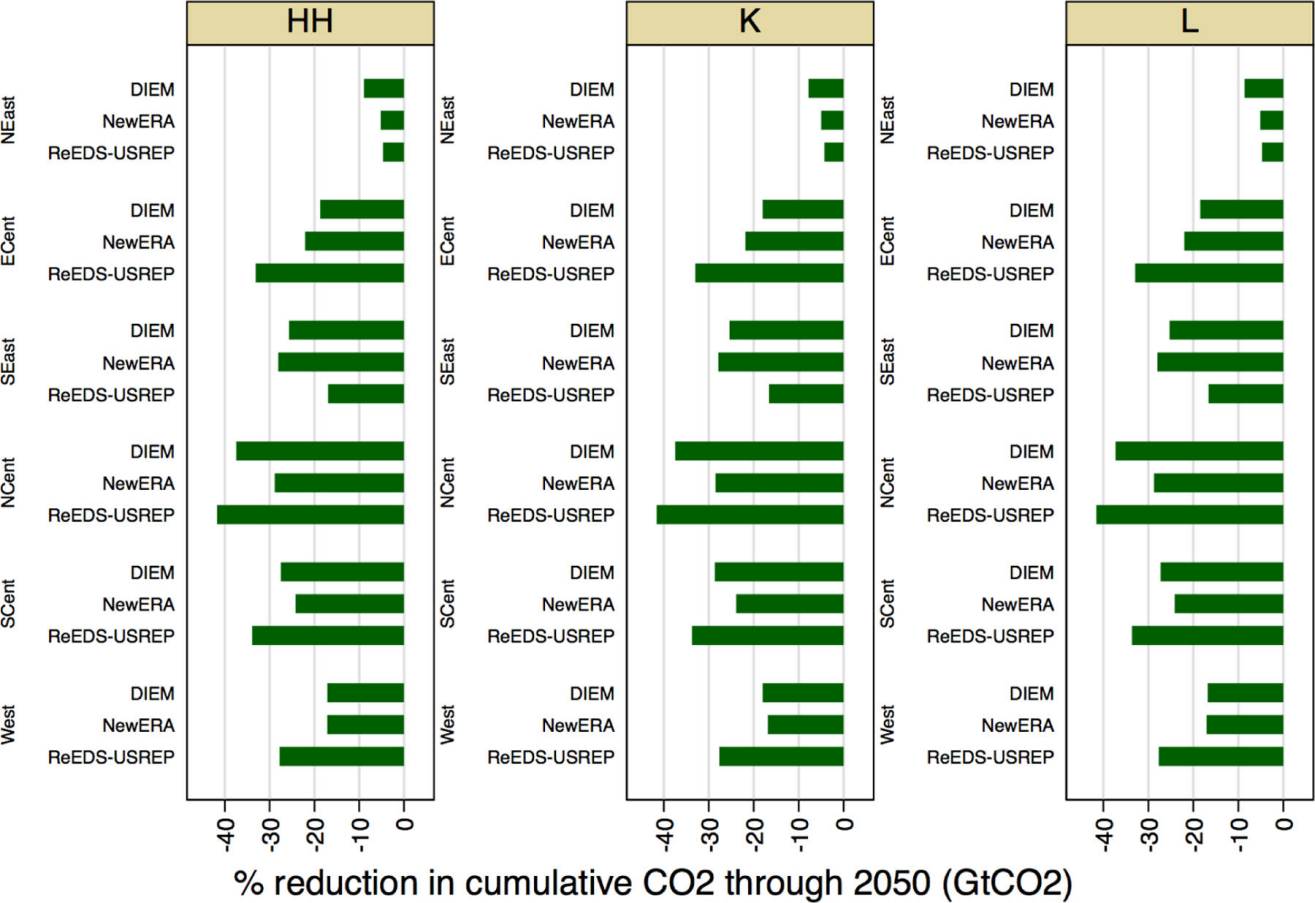
Percent reduction in cumulative CO_2_ through 2050 by region for a $25@5% tax.

We first look at the distribution of emissions reductions (abatement) across regions. In Fig. 14, we present percent cumulative emissions reductions relative to reference emissions through 2050 across U.S. regions for each model and for the core revenue recycling schemes. Figure A.6 in Appendix A displays corresponding reductions in absolute terms. There is much more variation between regions than across revenue recycling schemes. There is also general agreement regarding the geographical patterns of abatement. All three models show that the Northeast (NEast) reduces its emissions the least and that reductions are the greatest in the North Central region (NCent). The South Central (SCent) and South East (SEast) regions also experience large reductions in emissions.

**Figure 15:**
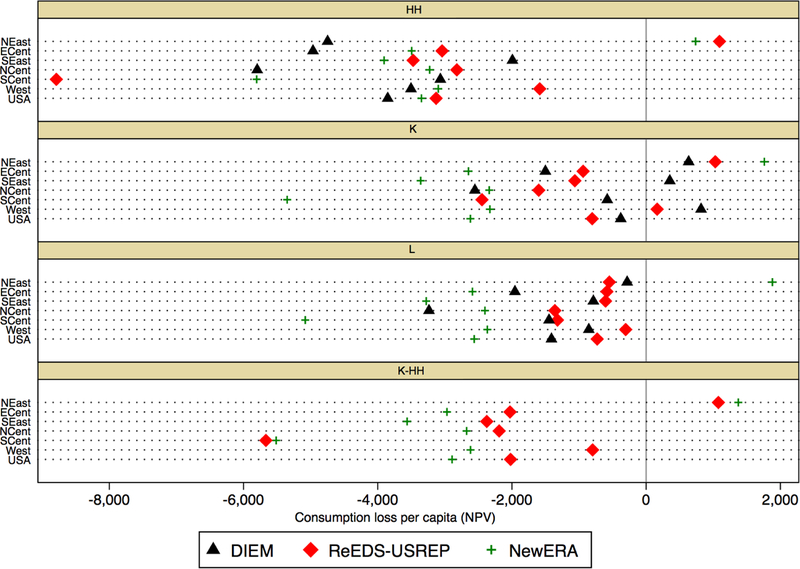
Consumption loss per capita (NPV) by region for a $25@5% tax.

Turning to the impacts on per capita consumption across regions in Fig. 15, two broad sets of observations emerge.

First, CO_2_ taxation will lead to substantial regional disparities in impacts with per capita consumption losses deviating substantially from the average “USA” value in all models and recycling schemes. The tax leads to reductions in the NPV of per capita consumption in most regions across the four revenue recycling schemes, with the exception of the Northeast in some schemes and models, and the West and the Southeast (SEast) in some models for capital recycling. These consumption gains are on the order of $1000. Per capita consumption losses are greatest in the South Central region across two of the models (ReEDS–USREP and NewERA) and in the North Central region in DIEM.

**Figure 16:**
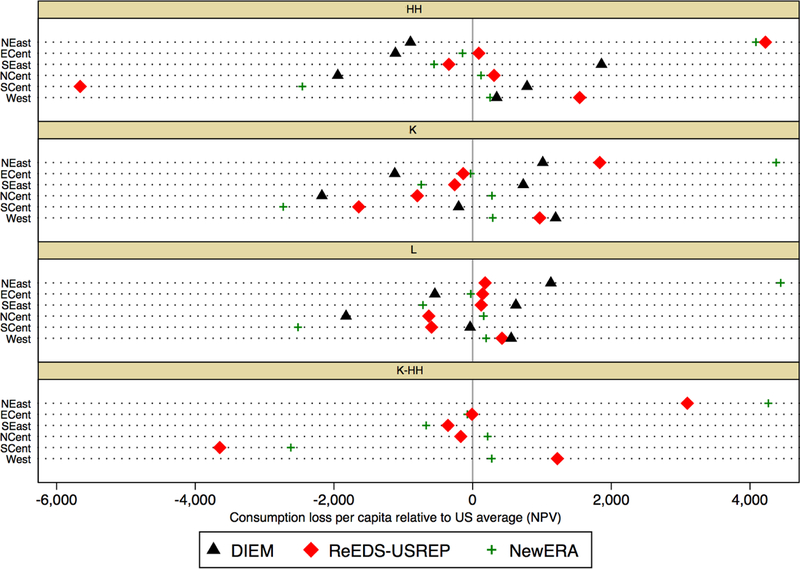
Regional consumption loss per capita (NPV), expressed relative to the average U.S. consumption loss, for a $25@5% tax.

Secondly, there is substantial variation across recycling schemes. These differences are even clearer in Fig. 16, in which we present results relative to average U.S. consumption loss for each scheme. Some findings here are robust across models: labor tax reductions on the whole seem to limit regional differences relative to HH and even K, for instance. Overall, however, the figure suggests relatively little agreement regarding which regions would profit more each recycling scheme.

Fully understanding what drives the regional disparities in impacts revealed by Fig. 15 is out of the scope of this study but in a relatively simple exercise, we investigate links between welfare impacts, income, and energy intensity.

**Figure 17:**
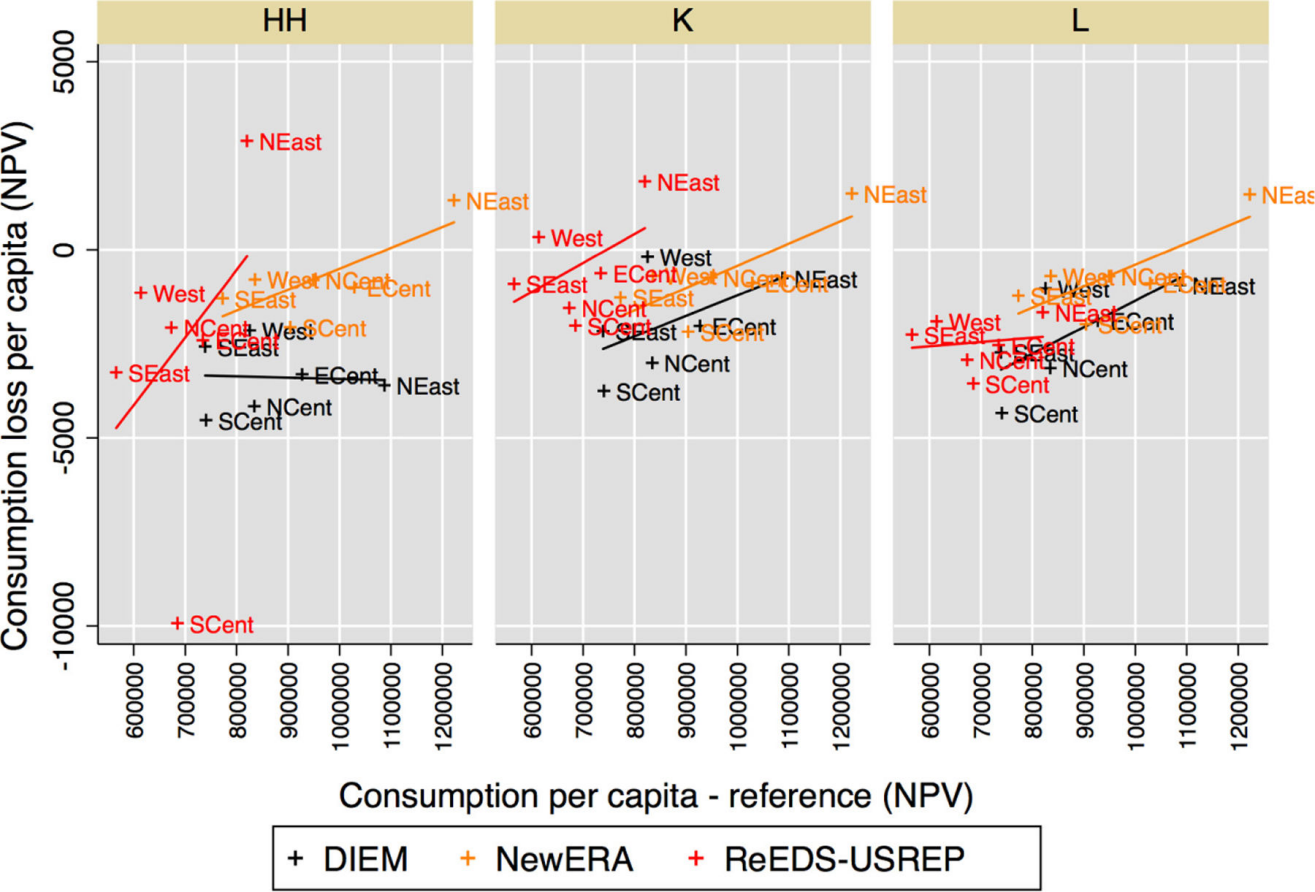
Consumption loss per capita (NPV) versus reference per-capita consumption, over regions, for a $25@5% tax. Lines represent a linear fit for each model.

We first look at whether the pattern of consumption losses corresponds to insights from the previous section on how regressive or progressive these policies are. In Fig. 17, we plot per capita consumption loss across regions against average regional per capita consumption in the reference case to determine whether there is a relationship between impacts and income within each model. One broad conclusion that is robust across the models is that capital tax reductions are regressive across regions and labor tax reductions somewhat less so, which is consistent with the results from the previous section. Lump-sum transfers (HH), however, do not seem to generally favor the lowest income regions, despite the fact that tax revenue is pooled at the national level before being re-distributed. This runs contrary to what we would expect from the results across income groups, although the results need not map directly on to each other, as income quintiles are obviously spread across regions (i.e., not all households in the lowest-income quintile are located in the region with the lowest per capita consumption).

**Figure 18:**
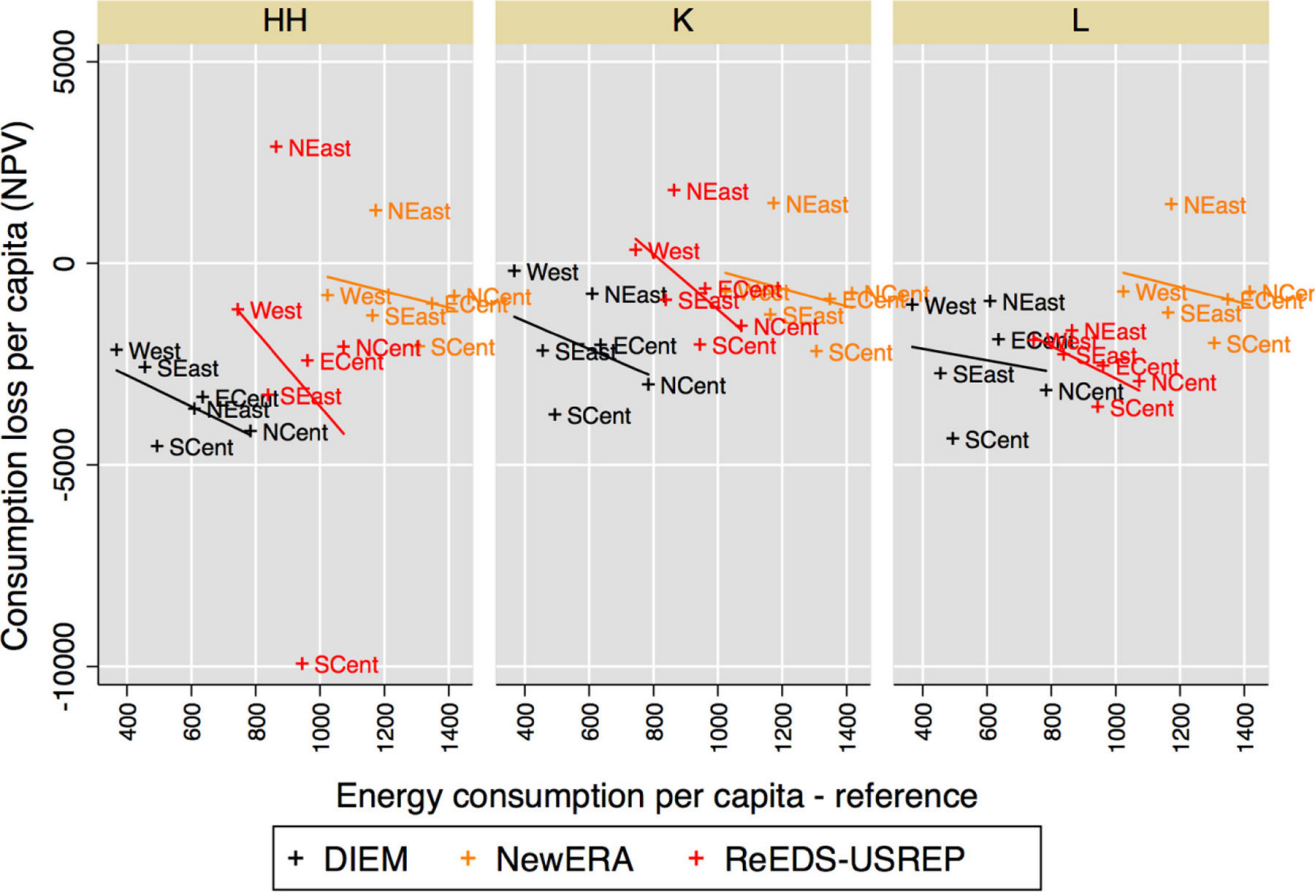
Consumption loss per capita (NPV) versus reference per-capita energy consumption over regions for a $25@5% tax. Lines represent a linear fit for each model.

We then plot per capita consumption loss against reference per-capita energy consumption in Fig. 18. Here, the models agree that regions with high per-capita energy consumption experience the largest decrease in consumption. The regions that have the largest reference energy consumption per capita also tend to be the regions that experience the largest consumption loss due to the need for greater emissions reductions in the presence of a CO_2_ price.

## 6. Conclusion

This paper provides the first multi-model comparison of the distributional impacts of carbon pricing. We confirm and expand upon two themes in the literature. First, the models agree on the ordering of the three core revenue recycling schemes in terms of efficiency and equity, and secondly, they agree that there is a trade-off between the two dimensions. In particular, revenue recycling in the form of capital tax reductions is the most efficient but the most regressive, whereas lump-sum rebates to households is the most progressive but the least efficient.

We also find that, given the large amount of revenue collected, equity considerations can be addressed and a large number of distributional outcomes are attainable to policy-makers through creative use of revenue. Going beyond the standard recycling schemes involving using all revenue toward either lump-sum rebates to households or capital tax reductions, our hybrid scenarios show that various points on the efficiency– equity frontier are attainable and that is even sometimes possible to improve on this frontier. Notably, we find that it is possible to protect low-income households with a modest share of revenues, while using the remainder of revenues on capital tax reductions allows the policy-maker to attain efficiency close to that of a pure capital tax reduction.

Though there is some disagreement on the magnitudes of consumption and welfare impacts, the degree of agreement regarding the above is notable given the very different assumptions underlying models. Some are recursive-dynamic, whereas some are forward-looking with fully intertemporally optimizing agents. Some are pure economic CGE models, whereas some are paired with detailed bottom-up representations of the electricity sector. Some have household types that are quite aggregated, while one model (IGEM) has hundreds of household types.

We see a number of avenues for research on the distributional impacts of carbon pricing moving forward. Future research should attempt to understand the assumptions driving the main differences between models, such as labor and capital supply elasticities, factor ownership patterns across households, and the role of indexed government transfers. The regional distribution of impacts is also a fruitful area for future research, as, in the present study, the models do not agree on the regional implications of the various revenue recycling schemes.

## Figures and Tables

**Table 1.** CO_2_ price paths and revenue recycling scenarios modeled.

CO_2_ price path	Revenue recycling option							
	HH	K	L	K-HH	TLQ-K	TLQ-L	TLQ-L-K	P-TLQ-K
$0	All 5 Models (DIEM, USREP-ReEDS, ADAGE, IGEM and NewERA)							
$25 at 1%	All 5 models	All 5 models	All 5 models	All except DIEM	USREP and ADAGE	USREP and ADAGE	USREP and ADAGE	USREP
$50 at 1%								
$25 at 5%								
$50 at 5%								

**Figure A.2:**
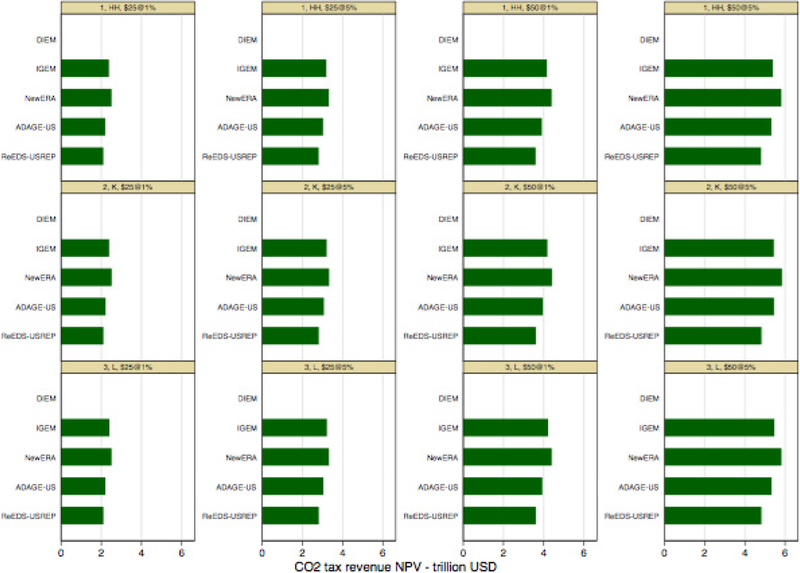
Large agreement regarding CO_2_ tax revenue across models, revenue recycling scheme, and CO_2_ price path.

**Figure A.3:**
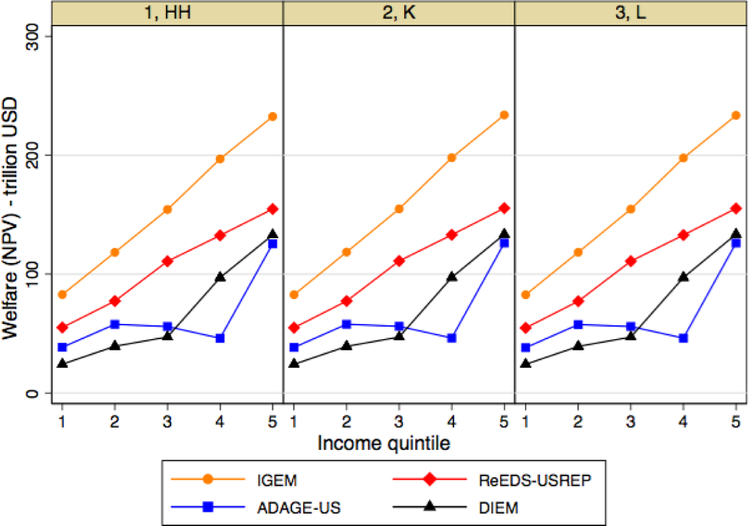
Distribution of welfare across income groups by model and revenue recycling scheme for $25 at 5% CO_2_ price path. Note that the distribution of welfare (levels) across models varies quite a bit. IGEM includes more leisure, explaining the larger differences between welfare and consumption impacts in that model. ADAGE has different patterns because it defines quintiles differently.

**Figure A.4:**
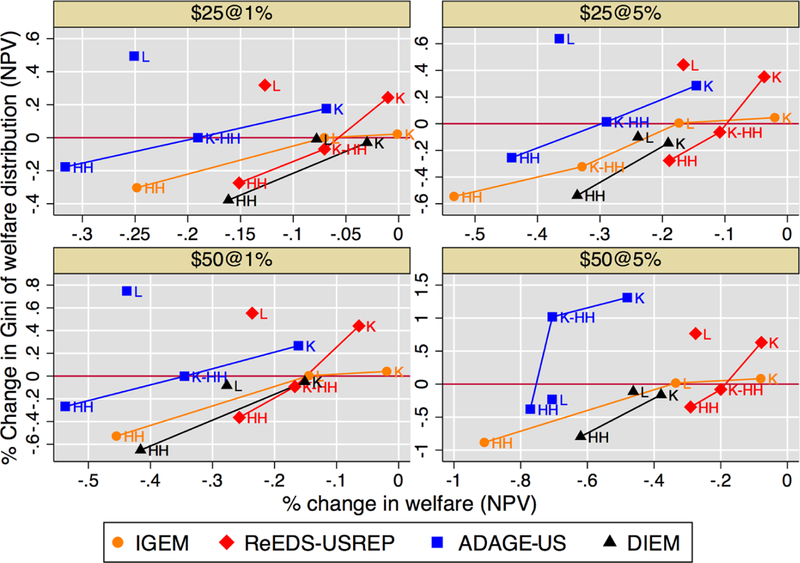
The trade-off between progressivity and aggregate welfare costs, with progressivity measured by the tax-driven change in the Gini coefficient of the welfare distribution. Note that axis scales differ across panels.

## Footnotes

1 Figure A.2 in the Appendix displays the NPV of the revenue collected by the taxes and shows that it is very similar across both models and revenue recycling schemes.

2 NewERA did not report reference welfare and therefore is not included in this figure.

3 Figure A.3. in the Appendix displays the corresponding distribution of welfare across the models.

4 A value *x*% on the *Y*-axis means that the recycling scheme makes the overall welfare distribution’s gini coefficient go up by *x*%.

## Appendix A.

**Table A.1.** Mappings for regional results.

Region	States
North East	ME, NH, VT, MA, CT, RI, NY
South East	KY, NC, TN, SC, GA, AL, MS, FL
East Central	DE, MD, PA, NJ, DC, VA, OH, WV
North Central	MO, ND, SD, MN, IA, WI, IL, MI, IN
South Central	OK, AR, LA, TX, NE, KS
West	MT, ID, WY, NV, UT, CO, AZ, NM, OR, WA, HI, CA, AK

**Table A.2.** NPV change in welfare (%) and consumption ($ per capita), 2020–2040, across models, tax trajectories, and revenue recycling options.

Model	$25@1%		$25@5%		$50@1%		$50@5%	
	Welfare (%)	Consumption ($ per cap)	Welfare (%)	Consumption ($ per cap)	Welfare (%)	Consumption ($ per cap)	Welfare (%)	Consumption ($ per cap)
DIEM								
HH	−0.16	−2726.39	−0.34	−3851.57	−0.42	−5513.47	−0.62	−8404.70
K	−0.03	135.40	−0.19	−378.63	−0.15	−599.44	−0.38	−2966.75
L	−0.08	−675.85	−0.24	−1411.39	−0.28	−2169.23	−0.46	−4529.81
IGEM								
HH	−0.25	−4420.65	−0.53	−5955.77	−0.45	−8344.43	−0.91	−10995.18
K	0.00	−2605.89	−0.02	−3676.33	−0.02	−5255.15	−0.08	−7241.27
L	−0.07	1871.32	−0.17	1481.44	−0.14	2282.29	−0.34	1377.22
K−HH			−0.33	−5152.14				
NewERA								
HH		−2770.65		−3753.51		−5365.10		−6812.81
K		−2138.02		−3087.51		−4293.56		−5722.76
L		−2147.62		−2862.20		−4270.87		−5290.87
K-HH		−2378.73		−3335.89		−4696.29		−6122.85
RTIADAGEUS								
HH	−0.32	−4402.53	−0.44	−5388.87	−0.54	−7314.27	−0.77	−9171.47
K	−0.07	−898.26	−0.15	−1661.52	−0.16	−1953.74	−0.48	−4978.32
L	−0.25	−2458.96	−0.37	−3124.85	− 0.44	−4355.00	−0.71	−5234.32
K-HH	−0.19	−2634.60	−0.29	−3494.77	−0.35	−4600.21	−0.71	−7901.32
TLQ-L-K	−0.20	−1977.83	−0.31	−2707.96	−0.36	−3613.84	−0.82	−5191.36
TLQ-L	−0.25	−2486.09	−0.37	−3160.88	− 0.44	−4395.62	−0.71	−5250.77
TLQ-K	−0.08	−1003.37	−0.17	−1844.49	−0.18	−2129.75	−0.58	−5817.76
ReEDS-USREP								
HH	−0.15	−2378.15	−0.19	−3201.82	−0.26	−4304.73	−0.29	−5423.18
K	−0.01	−269.77	−0.04	−864.07	−0.06	−1334.66	−0.08	−2063.43
L	−0.13	−270.23	−0.17	−742.97	−0.24	−1261.29	−0.28	−1852.47
K-HH	−0.07	−1263.66	−0.11	−2079.58	−0.17	−3058.33	−0.20	−4132.54
TLQ-L-K	−0.06	−445.79	−0.10	−1050.79	−0.15	−1674.25	−0.17	−2402.66
TLQ-L	−0.12	−527.18	−0.16	−1083.65	−0.23	−1767.07	−0.27	−2455.00
TLQ-K	−0.02	−416.11	−0.05	−1071.87	−0.08	−1675.75	−0.10	−2456.96
P-TLQ-K	−0.03	−580.56	−0.06	−1294.43	−0.10	−1980.58	−0.12	−2840.22

**Table A.3.** Aggregate welfare cost of neutralizing economic impacts on lowest income quintile households (% reduction in welfare cost relative to lump-sum transfers to household sin HH

	$25@1%		$25@5%		$50@1%		$50@5%	
	ADAGE-US	ReEDS-USREP	ADAGE-US	ReEDS-USREP	ADAGE-US	ReEDS-USREP	ADAGE-US	ReEDS-USREP
K	−78.3	−93.3	−67.0	−80.4	−69.9	−75.1	−37.7	−73.1
TLQ-K	−74.6	−87.5	−62.3	−73.9	−66.3	−67.2	−25.4	−64.7
P-TLQ-K		−80.9		−67.1		−60.1		−57.2
TLQ-L-K	−37.0	−58.0	−30.8	−48.9	−33.1	−43.3	5.9	−41.0
TLQ-L	−19.5	−18.3	−16.2	−14.4	−17.5	−10.2	−8.3	−7.7

**Table A.4.** Revenue requirements of neutralizing capital tax scenarios for lowest income quintile households (share of tax revenue recycled).

	$25@1%		$25@5%		$50@1%		$50@5%	
	ADAGE-US	ReEDS-USREP	ADAGE-US	ReEDS-USREP	ADAGE-US	ReEDS-USREP	ADAGE-US	ReEDS-USREP
P-TLQ-K		0.08		0.10		0.10		0.11
TLQ-K	0.09	0.07	0.11	0.09	0.08	0.09	0.29	0.10
TLQ-L	0.27	0.14	0.26	0.15	0.23	0.15	0.06	0.15
TLQ-L-K		0.10		0.11		0.11		0.12
